# Variation in Morphological and Quality Parameters in Garlic (*Allium sativum* L.) Bulb Influenced by Different Photoperiod, Temperature, Sowing and Harvesting Time

**DOI:** 10.3390/plants9020155

**Published:** 2020-01-26

**Authors:** Muhammad Jawaad Atif, Bakht Amin, Muhammad Imran Ghani, Muhammad Ali, Zhihui Cheng

**Affiliations:** 1 Department of Vegetable Science, College of Horticulture, Northwest A&F University, Yangling 712100, China; jawaadatif@nwafu.edu.cn (M.J.A.); bakhtamin96@nwafu.edu.cn (B.A.); imran_pak@nwsuaf.edu.cn (M.I.G.); muhammadali@nwsuaf.edu.cn (M.A.); 2 Vegetable Crops Program, National Agricultural Research Centre, Islamabad 44000, Pakistan; 3 College of Natural Resource and Environment, Northwest A&F University, Yangling 712100, China

**Keywords:** garlic cultivars, light, temperature, sowing date, plant age, bulb, soluble protein, sugars, total phenols, total flavonoids

## Abstract

Photoperiod (light) and temperature as abiotic factors having significant impact on the garlic bulb morphology and quality. In various bulb plants including garlic, bulbing is affected by photoperiod, temperature, sowing date and the plant age. In this backdrop experiments were performed to understand the effect of different photoperiods (10 h/14 h, 12 h/12 h and 14 h/10 h (light/dark)), temperatures (25 °C/18 °C and 30 °C/20 °C (light/dark)), sowing dates (D_0801_: 1^st^ August, D_0901_: 1^st^ September and D_1001_: 1^st^ October) and plant ages (A_80_, A_60_ and A_40_: 80, 60 and 40 days after planting) on garlic cultivars viz; G103, G024 and G2011-5. Parameters including morphological (plant height, fresh weight and pseudostem diameter), bulb attributes (diameter, weight, height and bulbing index), growth period and bulb quality related traits (total soluble solid (TSS), contents of soluble protein, soluble sugar, total sugar, glucose, sucrose, fructose, starch, total phenol and total flavonoid) were assayed. Longer photoperiod (14 h), higher temperature (30 °C), early sowing (D_0801_) and maximum plant age (A_80_) had maximum morphological and bulb quality related traits for cv. G103. These results showed that early sowing, maximum plant age, longer photoperiod and higher temperature are important for garlic bulb formation and quality. Moreover, the regulation of garlic bulb morphology and quality is achievable over the switch of sowing date, plant age, light and growth temperature.

## 1. Introduction

Garlic (*Allium sativum* L.) is the second most significant *Allium* crop. In addition to its regular use as a vegetable and condiment, it is known for its medicinal and nutraceutical properties. Growth of garlic mainly depends on the time of planting as the vegetative growth is stimulated under a short photoperiod and low temperature and bulb production is enhanced by a long photoperiod and high temperature. Sowing date and plant age significantly influence the various growth and developmental traits of garlic bulb [1,2,3]. Garlic is produced in most areas with mild winters and some rainfall, followed by dry summers [4,5]. Since all garlic cultivars are sterile, reproduction is generally vegetative with the cloves serving as propagation material [6]. Garlic bulb maturation is characterized by the drying up of foliage leaves and prior to harvest, the bulbs enter a period of dormancy when they exhibit almost complete inactivity regardless of environmental conditions with a low degree of respiration [4,7]. Several factors limit the rate of sprouting in garlic: cultivar, photoperiod, temperature and maturity at harvest [6,8]. Plant growth in terms of leaf number and plant height increases until bulbing is initiated. Ideally, plants should achieve adequate growth before bulbing begins, so that the foliage is capable to produce large bulbs and high yields [6]. Water and carbohydrates are the primary constituents of garlic bulbs, consisting of about 65% of its fresh weight and 77% of its dry weight, individually [9,10]. The key proportion of carbohydrates comprises of water-soluble high-molecular-weight fructose polymers called fructans [3]. Garlic cloves also incorporate proteins, pectin, minerals, and polyamines [11].

Bulb growth is affected mainly by genetics and environmental factors, especially photoperiod and growth temperatures, and the plant’s phenological stage [12,13,14,15]. Recent studies on *Arabidopsis*, rice, wheat, *Medicago*, onion and other plants have reported that environmental factors such as photoperiod, temperature and internal factors (plant age, gibberellic acid and sugars such as trehalose-6-phosphate (Tre6P)) regulate flowering through expression of the *FLOWERING LOCUS* T (FT) protein, which acts as a systemic floral signal molecule [16,17,18]. In *Allium*, different FT homologs have been attributed with control of bulbing [13,19]. Research on the effect of the photoperiod and temperature on garlic growth and bulb development provides an understanding of the influence of environmental factors [20]. Long photoperiod promotes early elongation of flower stalks and growth of bulbs, but exposure to long photoperiod results in a decrease of flower growth by variation in the development of topsets [20]. Photoperiod, like other ecological stimuli, controls plant growth over internal signals and variations in hormonal profile. A long photoperiod is known to increase endogenous gibberellins level, with the significant florogenesis [21]. Bulb growth is also enhanced by high temperatures [22], and develops earlier with a rise in temperature when a photoperiod is adequate [23]. Therefore, temperature might influence the growing proportion of plants and subsequent bulb growth. Caruso et al. [24] evaluated the properties of transplanting times on the yield of the cultivar ‘Ramata di Montoro’, which is a common cultivar in the Campania region of Italy, and specified that bulb size decreased considerably from the earliest to the latest transplanting time.

Phenolics are secondary metabolites in plants and play vital roles in the nutritional qualities of vegetables [25,26]. Phenolic compounds have extensive bioactive functions like antinutritional factors, phytoalexins, attractants for pollinators [27] and antioxidants [28], and can be used in the food industry as natural and potent food preservatives. There has been growing attention to improvements in the nutraceutical and economic values of crops. Favorable conditions might cause favorable changes in plant metabolites. It has been revealed that the bioactive components altered during sprouting. Isoflavone and oligosaccharide levels fluctuated [25], and stages of tocopherols, amino acids and carbohydrates gradually increased during sprouting [29]. Levels of vitamin C, total phenolics and total flavonoids increased in a time-dependent manner [30]. Light significantly affected synthesis of phenolics [31]. Flavonoids, important phenolic compounds, increased in abundance under light irradiance [32]. Light affected the properties of polysaccharides and increased associated antioxidant activities [33].

In China, garlic is consumed as a spice and as a seasonal vegetable. Garlic bulbs are considered a high-value vegetable throughout Asia and the demand for bulbs is increasing. Though, variations in climate, geography, photoperiod and temperature significantly alter the bulb production and quality. In addition, the influence of photoperiod and temperature variations on the development of garlic bulbs is poorly understood. One of the most significant limitations of the research of photoperiod and temperature in garlic is the difficulty in controlling for plant age. A systematic understanding of the photoperiod and temperature environments required for garlic growth would improve our knowledge of the bulbing process, facilitate the production of bulbs in different environments and geographies, and enrich our understanding of growth regulation in other bulbous plant species. As a result, year-round production of garlic may be possible. Experiments have been designed to evaluate the effect of different sowing dates and plant ages in combination with photoperiod and temperature regimes on garlic bulb morphology and quality parameters. 

## 2. Results

### 2.1. Effect of Cultivar, Sowing Date, Plant Age, Photoperiod and Temperature Treatments on Garlic Morphological Traits

The effect of each factor on garlic morphological traits was analyzed (Table 1 and Table 2). All of the studied factors, including cultivar (C), photoperiod (L), temperature (T), sowing date (D) and plant age (A) had a highly significant effect on plant height, fresh weight, pseudostem diameter, bulb characteristics (diameter, weight and height), bulbing index (BI) and growth period (Table 1 and Table 2). The longer photoperiod (14 h) and higher test temperature (30 °C) presented a significant enhancing effect on the BI, plant height, maturity and bulb characteristics of garlic among the treatments (Table 1 and Table 2). Cv. G024 had the highest BI, while cv. G103 produced the highest plant height, bulb characteristics and shortest growth period (Table 1 and Table 2). Early sowing date (D_0801_) and maximum plant age (A_80_) had a significant effect on all of the studied indicators (Table 1 and Table 2; Figures S1–S8).

### 2.2. Garlic Bulb Physiological and Nutritive Quality Traits Affected by Cultivar, Sowing Date, Plant Age, Photoperiod and Temperature

The effect of each factor on physiological and nutritive quality traits in garlic bulb was analyzed (Table 3 and Table 4). A significant influence was recorded for all of the studied factors (D, A, C, L and T). The responses were similar to BI and bulb characteristics (Table 3 and Table 4). Data analysis revealed maximum total soluble solid (TSS), contents of soluble protein, soluble sugar, total sugar, glucose, sucrose, fructose, starch, total phenol and total flavonoid were measured in long photoperiod (14 h), high temperature (30 °C), early sowing time (1^st^ August) and maximum plant age (80 days after planting) for cv. G103 (Table 3 and Table 4; Figures S9–S18).

## 3. Discussion

The results of our study showed that bulb characteristics and bulb quality related traits were higher at early sowing and maximum plant age (Table 1, Table 2, Table 3 and Table 4; Figures S1–S18). The response of varied sowing dates on bulb development and physiology is different [34]. Long photoperiod and high temperature are suitable for healthy bulb growth. Rahman and Talukda [35] have also reported the similar results. Ali and El-Said [36] evaluated the effect of different planting times and varieties on garlic bulb yield and quality attributes and the results exhibited that early planting resulted in maximum bulbs. In the present study bulbing reduced constantly with late sowing (Table 1 and Table 2; Figures S4–S7). The primary phenological stages in the garlic production are the number of days from sowing to emergence, emergence to bulb development, clove development, leafing, bulb initiation and maturity [37]. We observed similar results among the bulb characteristics. Sowing dates significantly influenced the growth and developmental traits of garlic (Table 1 and Table 2; Figures S1–S8). Garlic bulb growth was affected by varied dates of planting. Upsurge in bulb growth was stimulated in early sowing (Table 1 and Table 2; Figures S4–S7). The valuable effect on plant growth because of early sowing had been stated by Qaryouti and Kasarawi [38]. The garlic plant morphology and bulb quality traits have been observed in maximum quantity with early sowing in comparison with late sowing (Table 1, Table 2, Table 3 and Table 4; Figures S1–S18). This results in maximum vegetative growth and bulbing, ultimately the plants have enlarged bulbs. Similar results have been reported by Das et al. [39] and Rahim et al. [40]. The response of different planting dates and plant age on bulb growth was very different. Early planting and maximum plant age constantly increased the bulb characteristics while late planting and minimum plant age decreased the bulb characteristics. The length and girth of bulb was noted at maximum with the first sowing date (Table 1 and Table 2; Figures S1–S8). Early plantation also significantly increased the weight and size of bulb. Highest bulb weight might be due to adequate cool and dry climate, which probably increased the vegetative growth and size of bulb. Early plantation produced highest yield due to the large size production of bulb. Late planting reduced significantly the number of cloves and clove size. It might be due to the reason that the plant did not receive a long cool growth period, which was crucial for the development of the bulb as specified by Rahim [41]. On the basis of the above discussion we can assume that the early sowing, maximum plant age of garlic, long photoperiod and high temperature increased the bulbing potential.

According to Brewster [42], onion stops making leaf blades and transfers to the formation of bulb scales when bulb formation is initiated, and the development from leaf growth to bulb formation depends on both the photoperiod and temperature [43]. Moreover, high levels of photosynthetically active radiation combined with a long photoperiod increased both bulb development and final bulb size [44]. Bulb diameter and weight significantly enhanced with increasing photoperiod and temperature (Table 1 and Table 2; Figures S1–S8), and a positive effect was observed on garlic bulb formation and quality. Hence, these results recommended that plant growth and bulb development increased as a result of a long photoperiod and high temperature. The bulb formation is strongly influenced by the choice of the cultivar, which correlates with the local weather and location [24]. So, one of the most important features to obtain high production is selecting the cultivar, which is appropriate for the climate and environment of the area. Garlic bulb development is induced by the day length and under an ideal growth temperature, which may vary among garlic cultivars [15]. Thus, to grow healthy garlic bulbs, we have investigated the environmental effects on the characteristics of different commercially used garlic cultivars to evaluate the bulbing performance and bulb quality.

The significant interaction between the photoperiod and temperature resulted in the identification of a critical photoperiod and temperature for bulbing (Figures S1–S8). Photoperiod might affect the development of primary and secondary metabolites in plants. In *Arabidopsis*, the long photoperiod condition increases the contents of sucrose, vitamin C and other metabolites [45]. In our study, the phenolic compounds in the bulb of garlic increased at a long photoperiod and high temperature, but we did not observe this surge in the short photoperiod (Table 3 and Table 4; Figures S17 and S18). Similarly, sugars in garlic bulbs, relatively lower in short day photoperiod, also revealed a positive response to the long-day photoperiod and high temperature (Figures S11–S18). Garlic bulb under medium-light and long-light conditions had higher soluble protein content than under short-light conditions (Table 3 and Table 4; Figure S10). This is constant with the results in bulb characteristics (Table 1 and Table 2; Figures S1–S8). Thus, lengthening the light duration and increasing the temperature can improve the quality of garlic bulb according to our investigation. Talbott and Zeiger [46] proposed that light eminence might affect the translocation of carbohydrates, which may affect starch break-down. During a red light the decline of starch is detected, whereas blue light increased the absorption of this polysaccharide in plants [47]. Du Toit et al. [48] stated that mature bulbs of *Lachenalia* could have diverse forms of starch and soluble sugars throughout the growth period. Several periods increased absorption of starch in bulbs of ‘Rupert’ thanin ‘Ronina’, both cultured under white and blue light. This might specify that the plants of ‘Rupert’ were in a dissimilar developing phase, near to the flowering time, in comparison to the plants from other treatments [49,50]. Ohyama et al. [51] have demonstrated that giving the growing point of mother and new bulblets of tulip cultured in the open field not only increase but also a different kind of carbohydrates might vary. Our investigation provided novel evidence on what way the photoperiod and temperature influence the alterations in primary and secondary metabolites in garlic bulb (Table 3 and Table 4; Figures S9–S18).

In the present study it was observed that a longer photoperiod and higher temperature resulted in a higher amount of soluble protein, sugars and phenolic compounds could contribute to the nutritive quality traits as well as the defense mechanism against the environmental alterations. Increased levels of nutritional traits reflected that the bulbs grew at a longer photoperiod and higher temperatures owned not only developed phenolics but they also had higher quantities of protein and sugars (Table 3 and Table 4; Figures S9–S18), which upsurged the general antioxidant properties of these bulbs. Present results could be used as a valuable tool for producing garlic bulbs with higher nutritive quality during the growing season in diverse topographical atmospheres. Increased growing temperature, produced fruit with the higher phenolic contents [52]. Similarly, in the present study, the total phenolics and total flavonoids as well as soluble protein and sugars of garlic cultivars increased due to an increase in growth temperature (Table 3 and Table 4; Figures S9–S18). Previous studies have also linked increased phenol content with defense and plant growth protection [53,54]. 

Therefore, garlic bulbs produced at a longer photoperiod and higher temperatures have higher antioxidants, which is an optimistic nutritional enrichment. These possible health benefits of phenolic compounds depend on their interest and uptake, which in order are determined by their assembly plus their conjugation with added phenolics, consist of glycosylation/acylation, molecular size and solubility [55,56,57,58]. The phenolic compounds measured in the current investigation were similar to those detected in other studies [59,60]. As reported earlier [60], phenolic compounds in the garlic bulb, which contained mainly the total phenols and total flavonoids, are strongly affected by the plant genotype. Besides the genotypic dependence, the environmental variations also influence the quantity of phenolics [60]. Furthermore, Mpofu et al. [61] has reported that the environmental factors impart greater influence on the content of phenolics compared to genotypes. Though these researchers evaluated diverse genotypes in their study, the key likely reason could have been that these researchers collected their samples from diverse fields, where all the environmental factors including soil pH, temperature and rainfall were different. In comparison, currently we used controlled environments in which the only altering treatments were the photoperiod and temperature. The contents of phenolic compounds determined in the garlic cultivars in the current research were parallel to those detected in earlier investigations [62,63,64,65,66], establishing that the biosynthesis of phenolic compounds in plants and the garlic bulb was strongly affected by biotic and abiotic factors such as the photoperiod and temperature.

## 4. Materials and Methods 

### 4.1. Experimental Material Sowing Site Description

The experimental material (garlic cloves) was planted at the Horticultural Experimental Station (34° 16′ N, 108° 4′ E), Northwest A&F University, Yangling, Shaanxi Province, China in 2018–2019. The region had a temperate monsoon climate, with 12–14 °C of the annual average temperature. The experimental plants grown in the soil had chemical characteristics: pH 7.83 (1:1 soil: water), electrical conductivity 239.1 μS cm^−1^ (1:5 soil: water), available N (nitrogen) 56.32 mg kg^−1^, available P (phosphorus) 52.57 mg kg^−1^ and available K (potassium) 224.90 mg kg^−1^. The plot was deep ploughed before plantation. Afterward, each plot was incorporated with 1.5 kg ‘Pengdixin’ (organic matter ≥ 30%, N + P + K ≥ 4%, humic acid ≥ 20%, organic sylvite ≥ 5%; Zhengzhou, Henan Province, China), 0.25 kg Stanley compound fertilizer (21-10-11; Yimeng, Shandong Province, China) and 0.4 kg ammonium hydrogen carbonate (Hanzhong, Shaanxi Province, China) as a basal dose of the fertilizer before plantation. During the growth period, standard agronomic practices were used to maintain the experimental plants.

### 4.2. The Effect of the Sowing Date, Photoperiod and Temperature on Garlic Morphology and Bulb Physiology (Trial 1) 

The trial was a four-factor (sowing date × cultivar × photoperiod × temperature) complete randomized experiment. Uniform cloves of three garlic cultivars viz; G103, G024 and G2011-5 were purified with 0.6% Dacotech (75% chlorothalonil, Syngenta, Guangzhou, Guangdong Province, China). The difference in the selected cultivar was: G103 (growth period: 240 days, bulb skin color: white, mean number of cloves per bulb: 12–14, weight of fresh bulb: 45–55 g), G024 (growth period: 240 days, bulb skin color: purple, mean number of cloves per bulb: 11–12, weight of fresh bulb: 40–50 g), G2011-5 (growth period: 250 days, bulb skin color: purple, mean number of cloves per bulb: 12–14, weight of fresh bulb: 45–55 g).

Afterward, the experimental cloves were planted in the field on 1^st^ August, 1^st^ September and 1^st^ October 2018 (these dates corresponded to the sowing date treatments of D_0801_, D_0901_ and D_1001_, respectively) at 5 cm depth, 5 cm plant to plant spacing and 20 cm row to row spacing. The plot length and width were 1 m and 3 m, respectively. On 30^th^ January 2019, the experimental plants of garlic cultivars were transplanted into 15 cm × 15 cm pots and were cultured with organic substrate ‘Jiahui’ (20%–25% of organic matter, 8%–10% of humic acid, 6.5–6.8 of pH; Liaocheng, Shandong Province, China). Four uniform garlic plants were received by each pot, and for each replication, nine pots were used per treatment. Three replications were used for this trial. On 30^th^ January 2019, the experimental plants were conditioned for 48 h under 20 °C/18 °C (light/dark) and 80% RH to reduce transplanting shock, before treatment.

On 1^st^ February 2019, the experimental plants were exposed to different combinations of photoperiod (10 h/14 h, 12 h/12 h and 14 h/10 h (light/dark)) and temperature (25 °C/18 °C and 30 °C/20 °C (light/dark)) treatments in six separate growth chambers (Ningbo Jiangnan Instrument Factory, Zhejiang Province, China) with 70% RH (relative humidity) and 105 μmol m^−2^ s^−1^ PAR (photosynthetic active radiation). On 12^th^ March 2019, 40 days after photoperiod and temperature treatment, six plants from six pots from each treatment of each replication were sampled randomly. After measurement of morphological traits, the bulbs were rinsed with distilled water and dried with absorbent paper. The evenly mixed samples were then absorbed in liquid nitrogen for several seconds and stored at −80 °C in freezer until analysis of the physiological and nutritive quality traits. Three technical replicates were used for analysis.

### 4.3. Effect of Plant Age, Photoperiod and Temperature on Garlic Morphological and Physiological Traits (Trial 2) 

The trial was a four-factor (plant age × cultivar × photoperiod × temperature) complete randomized experiment. Uniform cloves of three garlic cultivars (G103, G024 and G2011–5) were selected as the experimental material. Garlic cloves were panted in the field on 20^th^ September, 10^th^ October and 1^st^ November, 2018, these dates corresponded to the plant age treatments. The cultural practices were same as in trial 1. On 8^th^ December 2018, thirty-eight days after the last planting date, plants at different ages were transplanted in pots (same organic substrate was used as in trial 1). 

On 8^th^ December 2018, the experimental plants were conditioned for 48 h under 20 °C/18 °C (light/dark) and 80% RH to decrease transplanting shock, before treatment. On 10^th^ December 2018, the experimental plants were exposed to different combinations of photoperiod (10 h/14 h, 12 h/12 h and 14 h/10 h (light/dark)) and temperature (25 °C/18 °C and 30 °C/ 20 °C (day/night)) treatments in six separate growth chambers (Ningbo Jiangnan Instrument Factory, Zhejiang Province, China) with 70% RH (relative humidity) and 105 μmol m^−2^ s^−1^ PAR (photosynthetic active radiation). The age of the plants at the time of photoperiod and temperature treatments was A_80_, 80 days after planting; A_60_, 60 days after planting; A_40_, 40 days after planting. Management practices were the same as that for the trial 1.

On 10^th^ March 2019, 90 days after the photoperiod and temperature treatment, six plants from six pots from each treatment of each replication were sampled randomly. After measurement of morphological traits, the bulbs were rinsed with distilled water and dried with absorbent paper. The evenly mixed samples were then absorbed in liquid nitrogen for numerous seconds and stored at –80 °C in a freezer until analysis of the physiological and nutritive quality indices. For analysis three technical replicates were used.

### 4.4. Assay of Garlic Morphological Indices

The garlic morphological traits were evaluated in the laboratory using a measuring tape (plant height), electronic balance (Changzhou, Jiangsu Province, China; fresh weight and bulb weight) and electronic Vernier caliper (Guanglu, Guilin Province, China; pseudostem diameter, bulb height and bulb diameter). The bulbing index (BI) was calculated as the ratio of the bulb diameter to pseudostem diameter. Bulbing was considered to start when BI = 2 [67]. Growth period (number of days) was calculated from the transplanting date (10 December and 01 February) in growth chambers to the harvest date.

### 4.5. Evaluation of Garlic Bulb Quality Related Traits

TSS (total soluble solid) content of the garlic bulb juice was measured by a digital refractometer (PAL-1, Minato-ku, Tokyo, Japan). The Coomassie Brilliant Blue (CBB) method was used to measure the soluble protein content [68]. Coomassie Brilliant Blue G-250 (100 mg) was dissolved in 50 mL 95% ethanol and to this solution 100 mL 85% (w/v) phosphoric acid was added. The resultant solution was diluted to a final volume of 1 liter. Final concentrations in the reagent were 0.01% (w/v) Coomassie Brilliant Blue G-250, 4.7% (w/v) ethanol and 8.5% (w/v) phosphoric acid. The absorbance was taken at 595 nm and content of protein was calculated from standard curve. 

Assessment of the soluble sugar content in garlic cloves was done according to Fei et al. [69]. The extracts of H_2_O were diluted with 80% methanol with a concentration of 10 mg/mL. One milliliter diluted extracts and 1.0 mL of 5% phenol were mixed and 5.0 mL H_2_SO_4_ was added and mixed carefully. After cooling absorbance was noted at 485 nm and concentration of soluble sugar was determined from the standard curve of glucose solution (concentration range from 0 to 100 µg/mL). Results were expressed as % of glucose equivalents for g of garlic cloves. Total sugar content was assessed according to McCready et al. [70]. A 500 mg sample was extracted in ethanol. After centrifugation at 5000× *g* for 10 min supernatant was mixed with 15 mL hydrochloric acid (1N) and incubated in water bath for 20 min. Afterward, 1 mL of hydrolyzed extract was taken in a test tube and 4 mL of anthrone reagent was added. After 10 min absorbance was read at 620 nm and calculations were done from standard curve of glucose.

Glucose content was determined according to the method of Miller [71]. Samples were extracted in distilled water and supernatant was mixed with 1 mL of alkaline copper tartrate reagent and the resulting solution was incubated in a boiling water bath for 10 min. The test tubes were removed and cooled followed by an addition of 1 mL of arsenomolybdate reagent. The volume of each test tube was made up to 10 mL with distilled water. After 10 min an absorbance reading was taken at a wavelength of 620 nm. The amount of reducing sugar present in the sample was calculated from the standard curve of glucose. Sucrose content was examined according to Handel, [72]. After extraction the supernatant was mixed with 2 N NaOH and incubated in the water bath for 10 min. After cooling, 3.5 mL hydrochloric acid (30%), and 1 mL resorcinol (0.1%) were added, and resulting solution was mixed thoroughly. After incubating at 80 ℃ in water bath for 30 min samples were cooled and read at 480 nm.

Measurement of fructose content was done according to Ashwell [73]. A 50 mg sample was extracted in 4 mL of 80% ethanol in a water bath at 80 ℃. Supernatant was collected and residue was again extracted in 2 mL of 80% ethanol. Ten milligrams of activated carbon was added to the supernatant to decolorize it. Two milliliters of 0.1% resorcinol and 1 mL of H_2_O were added and incubated at 80 ℃ in a water bath for 10 min. After cooling at room temperature OD (optical density) was read at 480 nm and calculation was done using standard curve of fructose. Starch content was assessed according to McCready et al. [70]. Residue left after sugar estimation was dried and extracted in 3 mL of water at 100 ℃ in a water bath for 20 min. Thereafter, 2 mL of 9.2 N perchloric acid was added and allowed to stand for 10 min followed by centrifugation at 4000× *g* for 15 min. After that 2.4 mL water and 1.6 mL of 9.2 N perchloric acid were added and allowed to stand for 10 min. Samples were again centrifuged and supernatants were mixed. To 0.4 mL extract was added 4 mL anthrone—sulfuric acid reagent and incubated at 90 ℃ for 15 min. After cooling absorbance was recorded at 620 nm.

An assessment of total phenol content was done according to Singleton and Rossi [74]. One hundred milligrams of the sample was extracted in methanol and the extract was centrifuged at 3000× *g* for 10 min. One milliliter of supernatant was mixed with 1 mL FC (Folin phenol) reagent and 3 mL of 20% Na_2_CO_3_. After 30 min absorbance was taken at 765 nm wavelength. A standard curve of gallic acid was used for calculation. The method of Yong et al. [75] was used to measure total flavonoids content. After extracting a 100 mg sample in 30% ethanol and 5 mL of 5% sodium nitrite was added to the supernatant and allowed to stand for 5 min. Afterwards 10% aluminum nitrite solution was added followed by the addition of sodium hydroxide. The absorbance was taken at 510 nm and standard curve of rutin was used for calculation.

### 4.6. Statistical Analysis

The data were analyzed using analysis of variance (ANOVA) as a 3 × 3 × 3 × 2 (sowing date × cultivar × photoperiod × temperature) factorial design for trial 1 and 3 × 3 × 3 × 2 (plant age × cultivar × photoperiod × temperature) factorial design for trial 2. SPSS 17.0 software (SPSS Inc., Chicago, IL, USA) was used for analysis. Tukey HSD (Honest Significant Difference) tests were used for separations of mean among treatments at *p* < 0.05. Three replicates were used for both trials.

## 5. Conclusions

In conclusion, the contents of the primary and secondary metabolites were significantly inclined by photoperiod, temperature, sowing date and plant age. Bulb characteristics reduced from the earliest to the latest sowing dates with an increase in photoperiod and temperature. Though, not only bulb growth but also the quality of the garlic bulb was affected by the photoperiod, temperature, sowing dates and plant ages. Variation in the sowing date, plant age, light (photoperiod) and temperature affected the soluble protein, sugars and phenolic compounds that affected the bulb development. Present research establishes a key foundation for selection of management and growing conditions from sowing to photoperiod and temperature regimes for better yield of garlic in varied environments. 

## Figures and Tables

**Table 1:** Effect of sowing date, cultivar, photoperiod and temperature treatments on garlic plant morphological traits, bulb characteristics, bulbing index and growth period.

Treatment	Plant Height (cm)	Fresh Weight (g)	Pseudostem Diameter (mm)	Bulb Diameter (mm)	Bulb Weight (g)	Bulb Height (mm)	Bulbing Index	Growth Period (Day)
Sowing date (dd.mm. y)								
01/08/2018	104.1 ± 14.7a	71.25 ± 16.05a	24.5 ± 5.4a	65.7 ± 7.8a	41.09 ± 5.75a	45.6 ± 5.8a	2.75 ± 0.37b	62.5 ± 12.9c
01/09/2018	88.1 ± 15.8b	48.35 ± 12.93b	19.6 ± 2.7b	56.0 ± 7.9b	32.42 ± 5.53b	41.5 ± 5.7b	2.87 ± 0.32a	68.6 ± 15.5b
01/10/2018	64.6 ± 13.8c	31.10 ± 10.22c	15.6 ± 1.9c	44.3 ± 6.5c	28.13 ± 5.53c	31.1 ± 8.8c	2.88 ± 0.52a	81.0 ± 23.1a
Cultivar								
G103	93.1 ± 20.8a	55.39 ± 20.78a	22.6 ± 7.5a	59.9 ± 10.5a	34.87 ± 7.60a	41.4 ± 9.0a	2.82 ± 0.55b	68.4 ± 18.5c
G024	73.5 ± 19.4c	44.78 ± 19.34c	17.4 ± 2.1c	49.9 ± 10.9c	34.55 ± 6.73b	37.4 ± 9.0c	2.85 ± 0.36a	71.3 ± 20.5b
G2011-5	90.2 ± 20.2b	50.54 ± 21.87b	19.8 ± 2.3b	56.2 ± 10.6b	32.23 ± 8.61c	39.4 ± 9.2b	2.83 ± 0.30b	72.3 ± 18.7a
Photoperiod (light/dark)								
10/14 h	74.3 ± 17.3c	36.69 ± 14.78c	18.8 ± 4.8c	50.1 ± 10.0c	29.12 ± 6.63c	32.9 ± 6.8c	2.72 ± 0.42c	91.7 ± 15.5a
12/12 h	83.6 ± 18.6b	50.35 ± 19.36b	19.8 ± 5.3b	54.5 ± 11.2b	33.12 ± 6.41b	38.7 ± 9.2b	2.82 ± 0.43b	67.0 ± 10.0b
14/10 h	99.0 ± 22.1a	63.66 ± 19.58a	21.1 ± 5.1a	61.4 ± 10.1a	39.40 ± 6.53a	46.6 ± 5.2a	2.97 ± 0.34a	53.4 ± 5.0c
Temperature (light/dark)								
25/18 °C	82.1 ± 21.1b	46.35 ± 21.25b	19.1 ± 4.3b	52.8 ± 10.7b	32.13 ± 7.72b	37.2 ± 9.1b	2.81 ± 0.43b	74.3 ± 19.8a
30/20 °C	89.1 ± 22.2a	54.12 ± 20.30a	20.7 ± 5.8a	57.8 ± 11.6a	35.63 ± 7.43a	41.6 ± 8.8a	2.86 ± 0.39a	67.0 ± 18.1b
*F*-test								
Sown date (D)	***	***	***	***	***	***	***	***
Cultivar (C)	***	***	***	***	***	***	***	***
Photoperiod (L)	***	***	***	***	***	***	***	***
Temperature (T)	***	***	***	***	***	***	***	***

Different letters indicate significant differences between means within columns at *p* < 0.05 by a Tukey HSD (Honest Significant Difference) test. *** *p* < 0.001, means ± SD.

**Table 2:** Effect of plant age, cultivar, photoperiod and temperature treatments on garlic plant morphological traits, bulb characteristics, bulbing index and growth period.

Treatment	Plant Height (cm)	Fresh Weight (g)	Pseudostem Diameter (mm)	Bulb Diameter (mm)	Bulb Weight (g)	Bulb Height (mm)	Bulbing Index	Growth Period (Day)
Plant Age (DAP)								
80	87.8 ± 12.0a	44.61 ± 8.25a	14.8 ± 2.0a	58.3 ± 6.9a	28.28 ± 4.08a	35.4 ± 4.7a	3.99 ± 0.53a	161.4 ± 28.6c
60	82.7 ± 11.2b	38.12 ± 6.14b	12.5 ± 1.8b	41.0 ± 5.8b	22.30 ± 3.78b	32.8 ± 4.6b	3.37 ± 0.49b	186.2 ± 35.4b
40	77.1 ± 10.6c	32.65 ± 4.06c	11.6 ± 1.7c	32.5 ± 4.7c	19.38 ± 3.72c	24.6 ± 6.7c	2.77 ± 0.35c	191.4 ± 29.1a
Cultivar								
G103	86.0 ± 10.1a	41.33 ± 8.61a	14.7 ± 2.1a	47.6 ± 12.3a	24.00 ± 5.23a	32.5 ± 7.0a	3.23 ± 0.66c	173.9 ± 26.5c
G024	80.1 ± 11.6c	36.07 ± 7.14c	11.3 ± 1.6c	39.6 ± 11.7c	23.77 ± 4.64b	29.4 ± 6.8c	3.50 ± 0.80a	180.5 ± 37.7b
G2011-5	81.5 ± 13.5b	37.99 ± 7.39b	12.9 ± 1.7b	44.5 ± 11.4b	22.18 ± 5.93c	30.8 ± 7.1b	3.41 ± 0.54b	184.5 ± 35.3a
Photoperiod (light/dark)								
10/14 h	70.6 ± 8.1c	33.45 ± 5.71c	12.6 ± 2.6b	38.8 ± 10.5c	19.31 ± 3.88c	25.4 ± 5.2c	3.07 ± 0.57c	215.9 ± 22.6a
12/12 h	84.8 ± 8.8b	37.31 ± 4.68b	12.6 ± 2.0b	43.7 ± 11.3b	23.58 ± 4.43b	30.5 ± 6,4b	3.47 ± 0.69b	176.1 ± 13.7b
14/10 h	92.2 ± 7.2a	44.62 ± 8.67a	13.7 ± 2.2a	49.3 ± 12.4a	27.07 ± 4.59a	36.9 ± 4.1a	3.59 ± 0.67a	146.9 ± 18.4c
Temperature (light/dark)								
25/18 °C	80.1 ± 13.0b	37.12 ± 6.63b	13.5 ± 2.3a	42.8 ± 12.3b	22.47 ± 5.31b	29.4 ± 7.0b	3.16 ± 0.63b	182.2 ± 32.0a
30/20 °C	84.9 ± 10.5a	39.81 ± 9.04a	12.5 ± 2.2b	45.0 ± 12.1a	23.29 ± 4.86a	32.5 ± 6.9a	3.59 ± 0.66a	177.1 ± 35.4b
*F*-test								
Plant Age (A)	***	***	***	***	***	***	***	***
Cultivar (C)	***	***	***	***	***	***	***	***
Photoperiod (L)	***	***	***	***	***	***	***	***
Temperature (T)	***	***	***	***	***	***	***	***

Different letters indicate significant differences between means within columns at *p* < 0.05 by a Tukey HSD (Honest Significant Difference) test. *** *p* < 0.001; DAP: days after planting, means ± SD.

**Table 3:** Effect of sowing date, cultivar, photoperiod and temperature treatments on garlic bulb physiological and nutritive quality traits.

Treatment	TSS (%)	Soluble Protein (mg g^−1^)	Soluble Sugar (%)	Total Sugar (mg g^−1^)	Glucose (%)	Sucrose (mg g^−1^)	Fructose (%)	Starch (mg g^−1^)	Total Phenol (mg g^−1^)	Total Flavonoid (mg g^−1^)
Sown date (dd.mm. y)										
01/08/2018	24.68 ± 4.98a	10.75 ± 0.63a	21.96 ± 6.33a	10.67 ± 3.09a	58.59 ± 15.61a	53.42 ± 13.14a	51.43 ± 20.35a	24.66 ± 7.00a	40.34 ± 15.93a	2.04 ± 0.59a
01/09/2018	23.03 ± 4.73b	8.42 ± 0.90b	20.82 ± 6.02b	10.16 ± 2.94b	46.79 ± 12.76b	42.70 ± 10.60b	39.88 ± 16.45b	21.59 ± 7.97b	35.57 ± 13.82b	1.62 ± 0.50b
01/10/2018	21.18 ± 4.48c	4.11 ± 0.76c	19.07 ± 6.97c	9.85 ± 2.96c	40.60 ± 11.30c	39.36 ± 9.50c	34.00 ± 14.86c	18.19 ± 5.70c	31.44 ± 13.30c	1.35 ± 0.36c
Cultivar										
G103	27.10 ± 4.13a	8.05 ± 2.78a	21.33 ± 6.33a	10.51 ± 3.00a	53.25 ± 16.15a	50.52 ± 11.29a	48.84 ± 20.37a	26.89 ± 7.59a	50.67 ± 12.48a	2.05 ± 0.51a
G024	18.42 ± 2.65c	7.46 ± 2.87c	19.93 ± 6.66c	9.92 ± 3.01c	43.16 ± 14.40c	39.34 ± 12.08c	31.42 ± 15.04c	17.22 ± 5.05c	23.47 ± 6.95c	1.13 ± 0.27c
G2011-5	23.37 ± 3.39b	7.77 ± 2.89b	20.59 ± 6.60b	10.25 ± 3.02b	49.58 ± 13.46b	45.61 ± 12.11b	45.05 ± 15.93b	20.34 ± 5.83b	33.20 ± 8.81b	1.83 ± 0.42b
Photoperiod (light/dark)										
10/14 h	20.26 ± 3.64c	7.26 ± 2.92c	14.25 ± 5.68c	7.18 ± 1.85c	35.61 ± 8.34c	34.70 ± 7.07c	28.97 ± 11.18c	16.69 ± 4.96c	27.79 ± 9.86c	1.44 ± 0.44c
12/12 h	22.65 ± 4.50b	7.63 ± 2.71b	21.31 ± 3.72b	10.46 ± 2.42b	49.69 ± 12.50b	46.68 ± 11.33b	38.22 ± 13.16b	20.23 ± 4.96b	35.85 ± 13.62b	1.62 ± 0.56b
14/10 h	25.98 ± 4.84a	8.39 ± 2.81a	26.29 ± 3.10a	13.05 ± 0.88a	60.69 ± 12.63a	54.09 ± 10.64a	58.11 ± 17.92a	27.52 ± 7.47a	43.70 ± 15.89a	1.94 ± 0.58a
Temperature (light/dark)										
25/18 °C	21.64 ± 4.29b	7.48 ± 2.93b	17.55 ± 7.13b	8.94 ± 3.16b	46.55 ± 15.64b	41.97 ± 12.67b	35.11 ± 15.81b	20.05 ± 6.43b	31.64 ± 12.00b	1.58 ± 0.52b
30/20 °C	24.29 ± 5.20a	8.04 ± 2.76a	23.69 ± 4.04a	11.52 ± 2.22a	50.78 ± 14.63a	48.34 ± 11.89a	48.43 ± 19.23a	22.92 ± 8.07a	39.92 ± 16.21a	1.75 ± 0.61a
*F*-test										
Sown date (D)	***	***	***	***	***	***	***	***	***	***
Cultivar (C)	***	***	***	***	***	***	***	***	***	***
Photoperiod (L)	***	***	***	***	***	***	***	***	***	***
Temperature (T)	***	***	***	***	***	***	***	***	***	***

Different letters indicate significant differences between means within columns at *p* < 0.05 by a Tukey HSD (Honest Significant Difference) test. *** *p* < 0.001, means ± SD.

**Table 4:** Effect of plant age, cultivar, photoperiod and temperature treatments on garlic bulb physiological and nutritive quality traits.

Treatment	TSS (%)	Soluble Protein (mg g^−1^)	Soluble Sugar (%)	Total Sugar (mg g^−1^)	Glucose (%)	Sucrose (mg g^−1^)	Fructose (%)	Starch (mg g^−1^)	Total Phenol (mg g^−1^)	Total Flavonoid (mg g^−1^)
Plant Age (DAP)										
80	18.31 ± 3.50a	7.07 ± 2.58a	20.04 ± 7.58a	9.99 ± 4.53a	60.37 ± 15.94a	18.28 ± 2.03a	49.00 ± 22.41a	30.34 ± 9.05a	26.73 ± 12.02a	1.52 ± 0.63a
60	17.28 ± 3.43b	6.60 ± 2.63b	18.94 ± 7.19b	9.46 ± 4.32b	48.19 ± 12.83b	17.02 ± 1.91b	40.43 ± 19.21b	24.72 ± 7.05b	23.07 ± 10.14b	1.40 ± 0.54b
40	15.91 ± 3.37c	6.14 ± 2.55c	16.69 ± 8.15c	9.07 ± 4.32c	41.38 ± 11.40c	16.17 ± 1.82c	33.14 ± 16.03c	21.39 ± 6.26c	20.01 ± 9.53c	1.22 ± 0.46c
Cultivar										
G103	20.39 ± 3.09a	6.86 ± 2.54a	19.48 ± 7.58a	9.86 ± 4.37a	53.30 ± 16.60a	18.35 ± 1.79a	48.81 ± 19.80a	29.44 ± 8.64a	32.02 ± 9.28a	1.85 ± 0.42a
G024	14.43 ± 1.99c	6.33 ± 2.63c	17.70 ± 7.81c	9.13 ± 4.42c	47.78 ± 16.23c	15.91 ± 1.97c	31.84 ± 17.50c	21.31 ± 6.73c	13.13 ± 6.18c	0.84 ± 0.21c
G2011-5	16.69 ± 2.58b	6.61 ± 2.65b	18.49 ± 7.84b	9.53 ± 4.42b	48.86 ± 13.34b	17.21 ± 1.80b	41.91 ± 20.26b	25.70 ± 7.73b	24.67 ± 7.45b	1.45 ± 0.45b
Photoperiod (light/dark)										
10/14 h	15.16 ± 2.60c	3.86 ± 1.71c	9.28 ± 3.26c	4.74 ± 2.28c	36.11 ± 8.05c	15.98 ± 1.89c	20.80 ± 8.95c	18.86 ± 4.21c	15.73 ± 7.06c	1.09 ± 0.42c
12/12 h	16.67 ± 2.98b	7.03 ± 1.82b	20.00 ± 3.68b	9.58 ± 2.42b	51.99 ± 13.01b	16.90 ± 1.68b	42.56 ± 14.15b	24.58 ± 5.48b	24.28 ± 9.49b	1.37 ± 0.46b
14/10 h	19.68 ± 3.45a	8.90 ± 1.15a	26.39 ± 2.83c	14.21 ± 1.55a	61.84 ± 12.75a	18.59 ± 1.85a	59.20 ± 15.23a	33.01 ± 7.90a	29.81 ± 10.96a	1.71 ± 0.60a
Temperature (light/dark)										
25/18 °C	16.80 ± 3.52b	5.77 ± 2.47b	17.56 ± 7.89b	8.73 ± 4.49b	45.56 ± 14.10c	16.82 ± 2.19b	38.22 ± 18.34b	23.84 ± 7.67b	21.09 ± 10.35b	1.27 ± 0.49b
30/20 °C	17.54 ± 3.58a	7.43 ± 2.49a	19.55 ± 7.54a	10.29 ± 4.19a	54.39 ± 15.86a	17.49 ± 1.97a	43.49 ± 22.04a	27.12 ± 8.77a	25.45 ± 11.13a	1.49 ± 0.60a
*F*-test										
Plant Age (A)	***	***	***	***	***	***	***	***	***	***
Cultivar (C)	***	***	***	***	***	***	***	***	***	***
Photoperiod (L)	***	***	***	***	***	***	***	***	***	***
Temperature (T)	***	***	***	***	***	***	***	***	***	***

Different letters indicate significant differences between means within columns at *p* < 0.05 by a Tukey HSD (Honest Significant Difference) test. *** *p* < 0.001; DAP: days after planting, means ± SD.

## Supplementary Materials

The following are available online at https://www.mdpi.com/2223-7747/9/2/155/s1, Figure S1. Plant height (cm); Figure S2. Fresh weight (g); Figure S3. Pseudostem diameter (mm); Figure S4. Bulb diameter (mm); Figure S5. Bulb weight (g); Figure S6. Bulb height (mm); Figure S7. Bulbing index; Figure S8. Growth period (day); Figure S9. TSS (%); Figure S10. Soluble protein content (mg g^−1^); Figure S11. Soluble sugar content (%); Figure S12. Total sugar content (mg g^−1^); Figure S13. Glucose content (%); Figure S14. Sucrose content (mg g^−1^); Figure S15. Fructose content (%); Figure S16. Starch content (mg g^−1^); Figure S17. Total phenol content (mg g^−1^); Figure S18. Total flavonoid content (mg g^−1^)
