# Supplementary material for: Variation in Morphological and Quality Parameters in Garlic (Allium sativum L.) Bulb Influenced by Different Photoperiod, Temperature, Sowing and Harvesting Time
Source: Plants (Basel). 2020 Jan 26;9(2):155. doi: 10.3390/plants9020155 (PMC7076542; doi:10.3390/plants9020155)
Supplement: Supplementary file 1 [file plants-09-00155-s001.pdf]

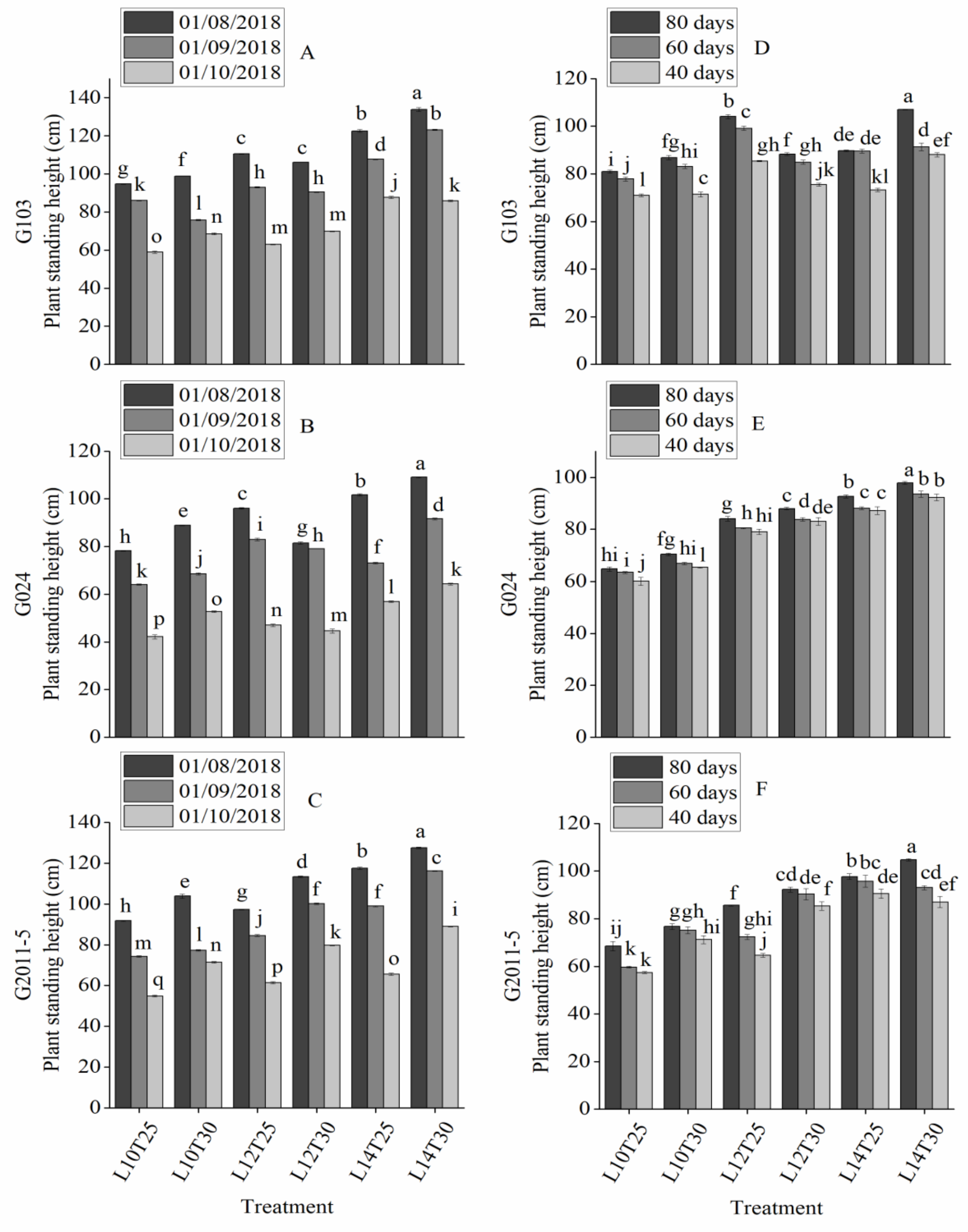

Figure S1. Plant standing height (cm) of cvs. G103 (A), G024 (B) and G2011-5 (C) sown on 1st August, 1st September and 1st October; cvs. G103 (D), G024 (E) and G2011-5 (F)- 80 days after planting, 60 days after planting and 40 days after planting. Different letters indicate significant differences between sowing date, plant age, photoperiod and temperature at $P<0.05$ (ANOVA and Tukey HSD test); means±SD

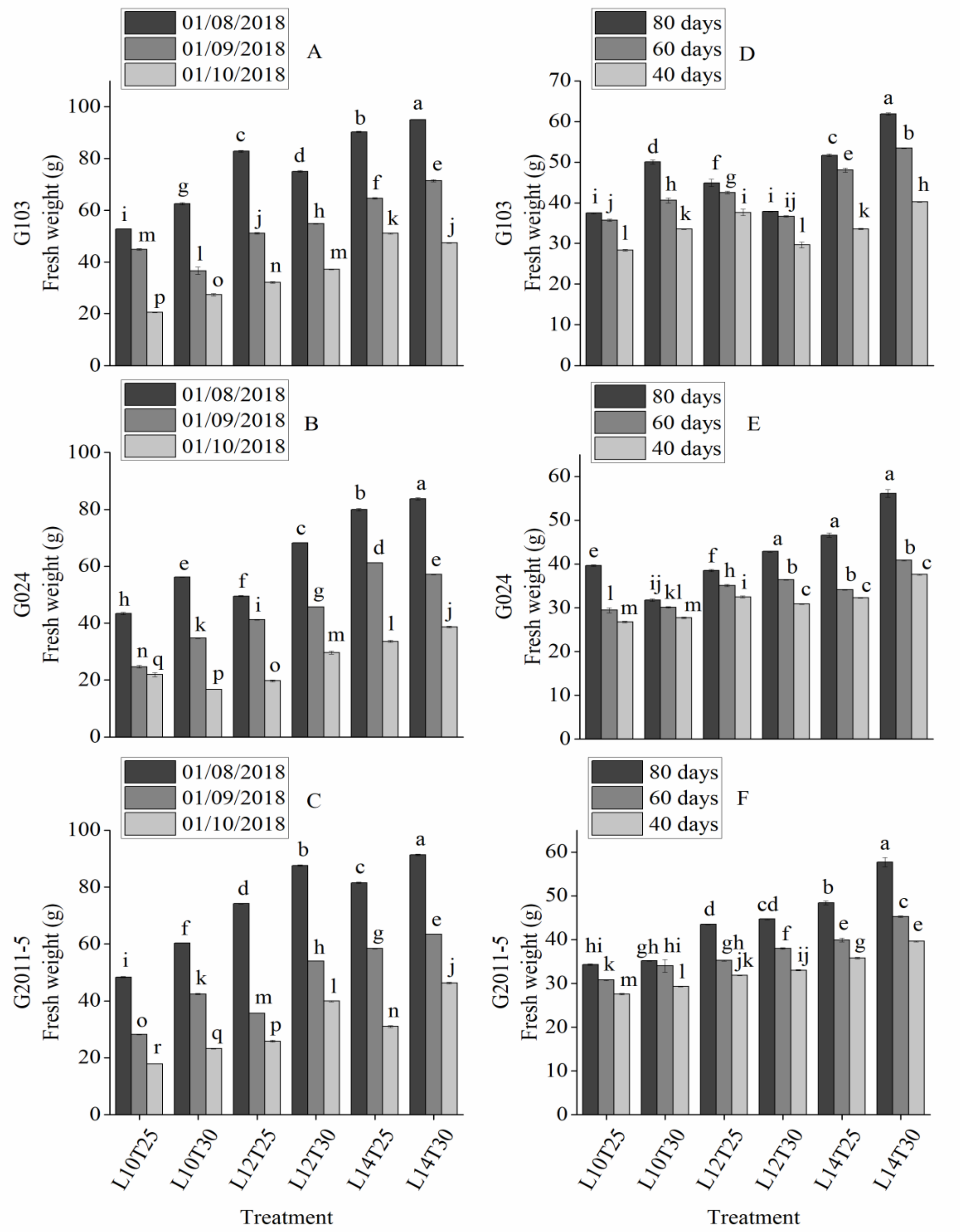

Figure S2. Fresh weight (g) of cvs. G103 (A), G024 (B) and G2011-5 (C) sown on 1st August, 1st September and 1st October; cvs. G103 (D), G024 (E) and G2011-5 (F)- 80 days after planting, 60 days after planting and 40 days after planting. Different letters indicate significant differences between sowing date, plant age, photoperiod and temperature at *P*<0.05 (ANOVA and Tukey HSD test); means±SD

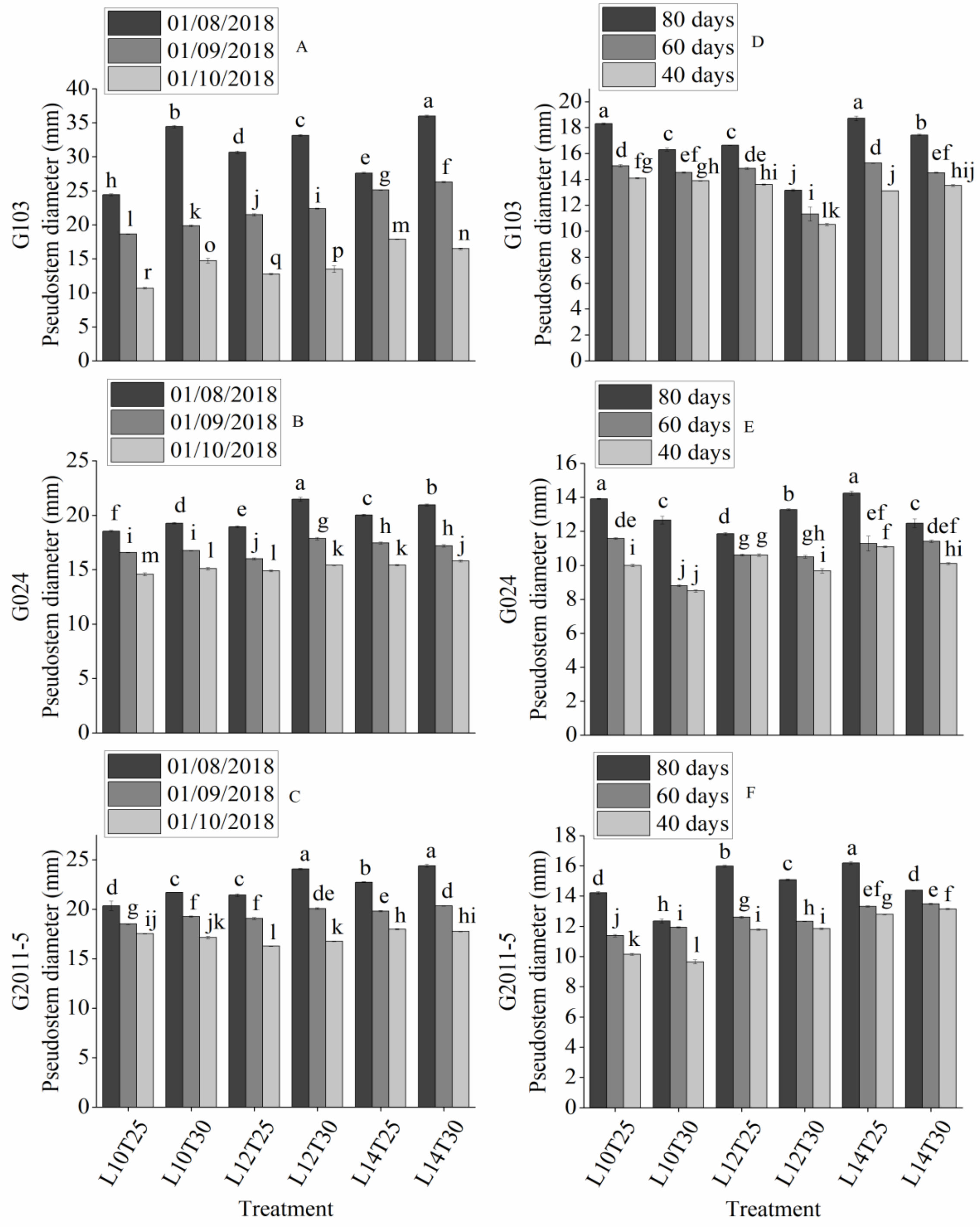

Figure S3. Pseudostem diameter (mm) of cvs. G103 (A), G024 (B) and G2011-5 (C) sown on 1st August, 1st September and 1st October; cvs. G103 (D), G024 (E) and G2011-5 (F)- 80 days after planting, 60 days after planting and 40 days after planting. Different letters indicate significant differences between sowing date, plant age, photoperiod and temperature at *P*<0.05 (ANOVA and Tukey HSD test); means±SD

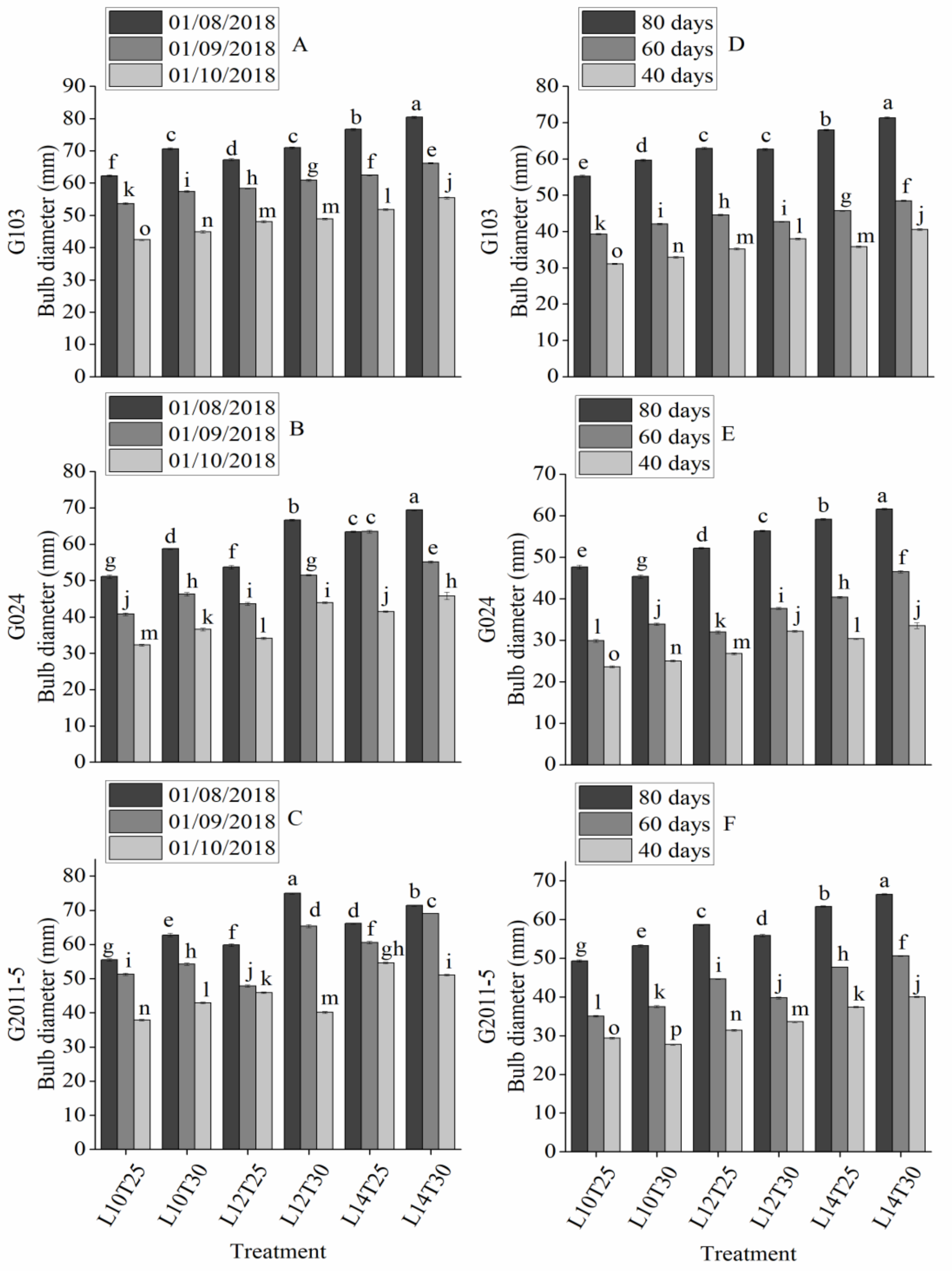

Figure S4. Bulb diameter (mm) of cvs. G103 (A), G024 (B) and G2011-5 (C) sown on 1st August, 1st September and 1st October; cvs. G103 (D), G024 (E) and G2011-5 (F)- 80 days after planting, 60 days after planting and 40 days after planting. Different letters indicate significant differences between sowing date, plant age, photoperiod and temperature at *P*<0.05 (ANOVA and Tukey HSD test); means±SD

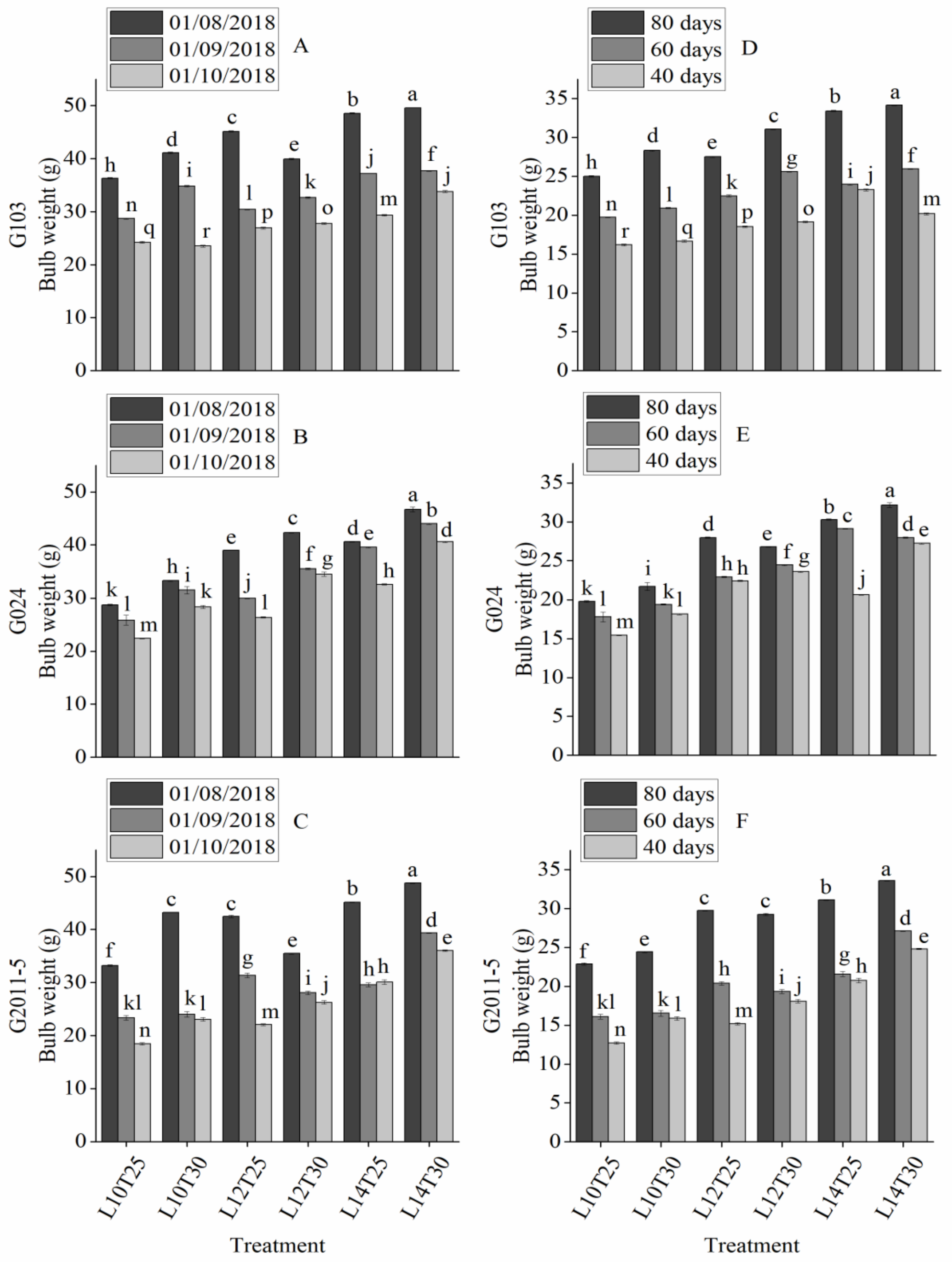

Figure S5. Bulb weight (g) of cvs. G103 (A), G024 (B) and G2011-5 (C) sown on 1st August, 1st September and 1st October; cvs. G103 (D), G024 (E) and G2011-5 (F)- 80 days after planting, 60 days after planting and 40 days after planting. Different letters indicate significant differences between sowing date, plant age, photoperiod and temperature at $P<0.05$ (ANOVA and Tukey HSD test); means±SD

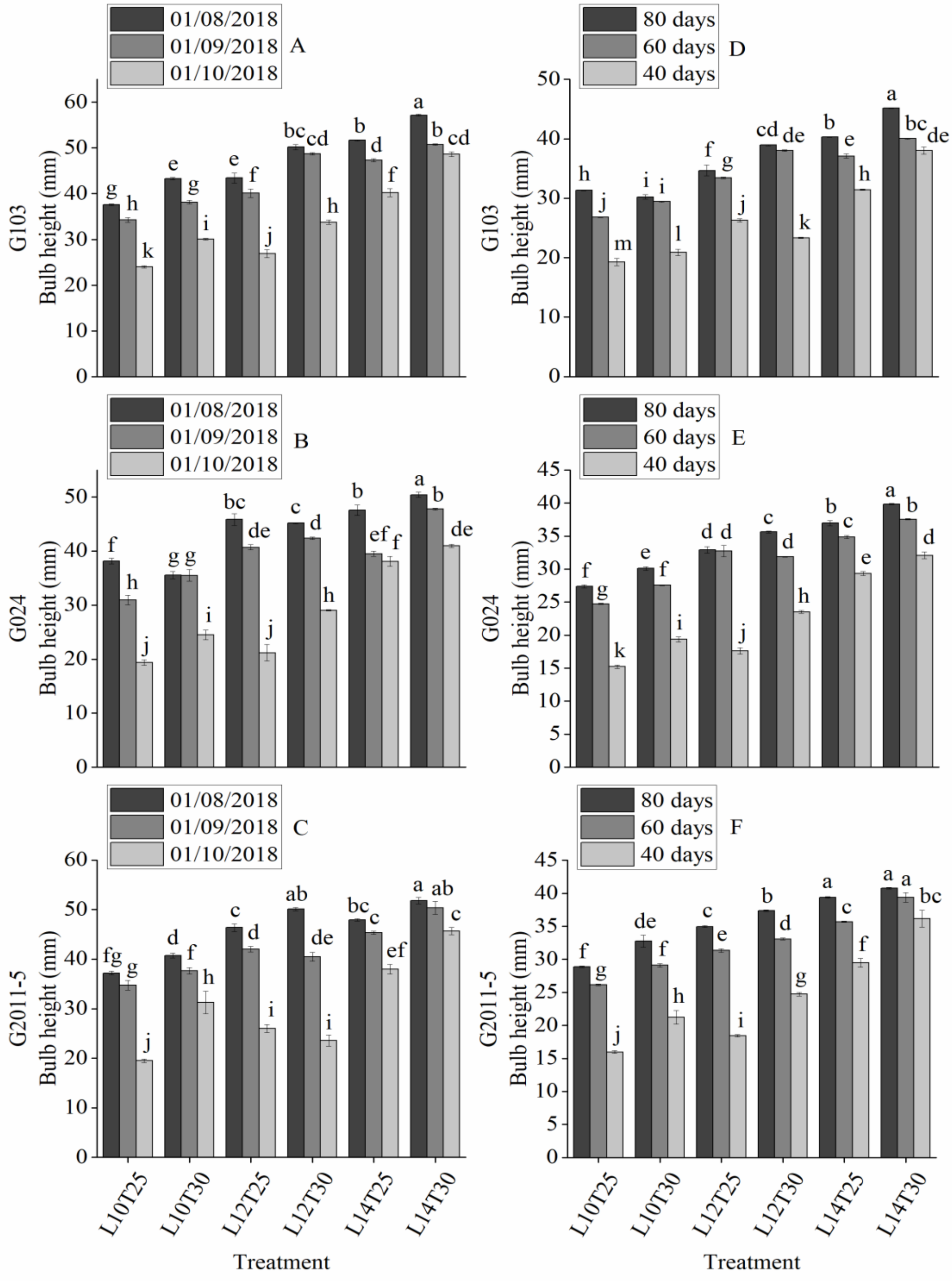

Figure S6. Bulb height (mm) of cvs. G103 (A), G024 (B) and G2011-5 (C) sown on 1st August, 1st September and 1st October; cvs. G103 (D), G024 (E) and G2011-5 (F)- 80 days after planting, 60 days after planting and 40 days after planting. Different letters indicate significant differences between sowing date, plant age, photoperiod and temperature at $P<0.05$ (ANOVA and Tukey HSD test); means±SD

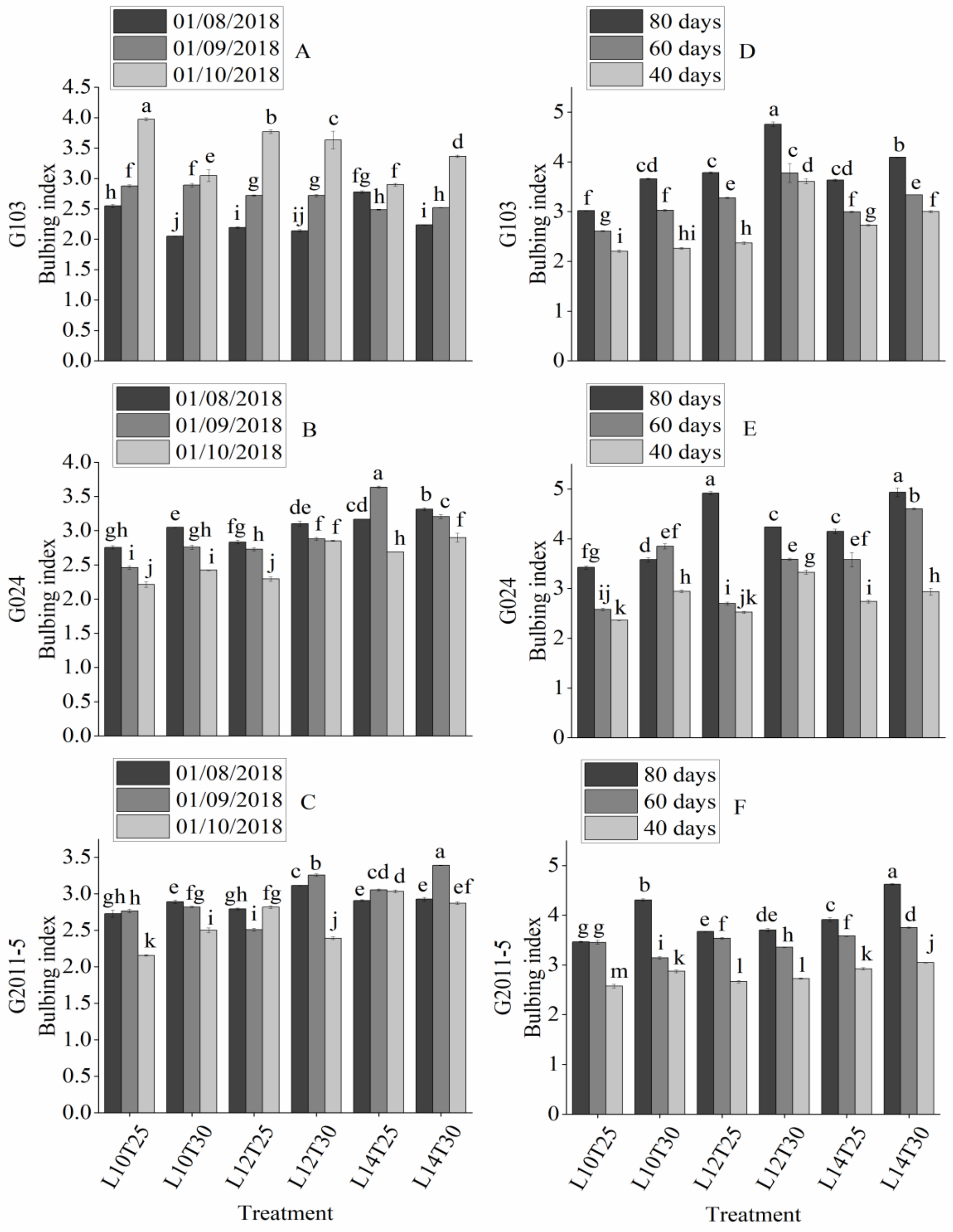

Figure S7. Bulbing index of cvs. G103 (A), G024 (B) and G2011-5 (C) sown on 1st August, 1st September and 1st October; cvs. G103 (D), G024 (E) and G2011-5 (F)- 80 days after planting, 60 days after planting and 40 days after planting. Different letters indicate significant differences between sowing date, plant age, photoperiod and temperature at *P*<0.05 (ANOVA and Tukey HSD test); means±SD

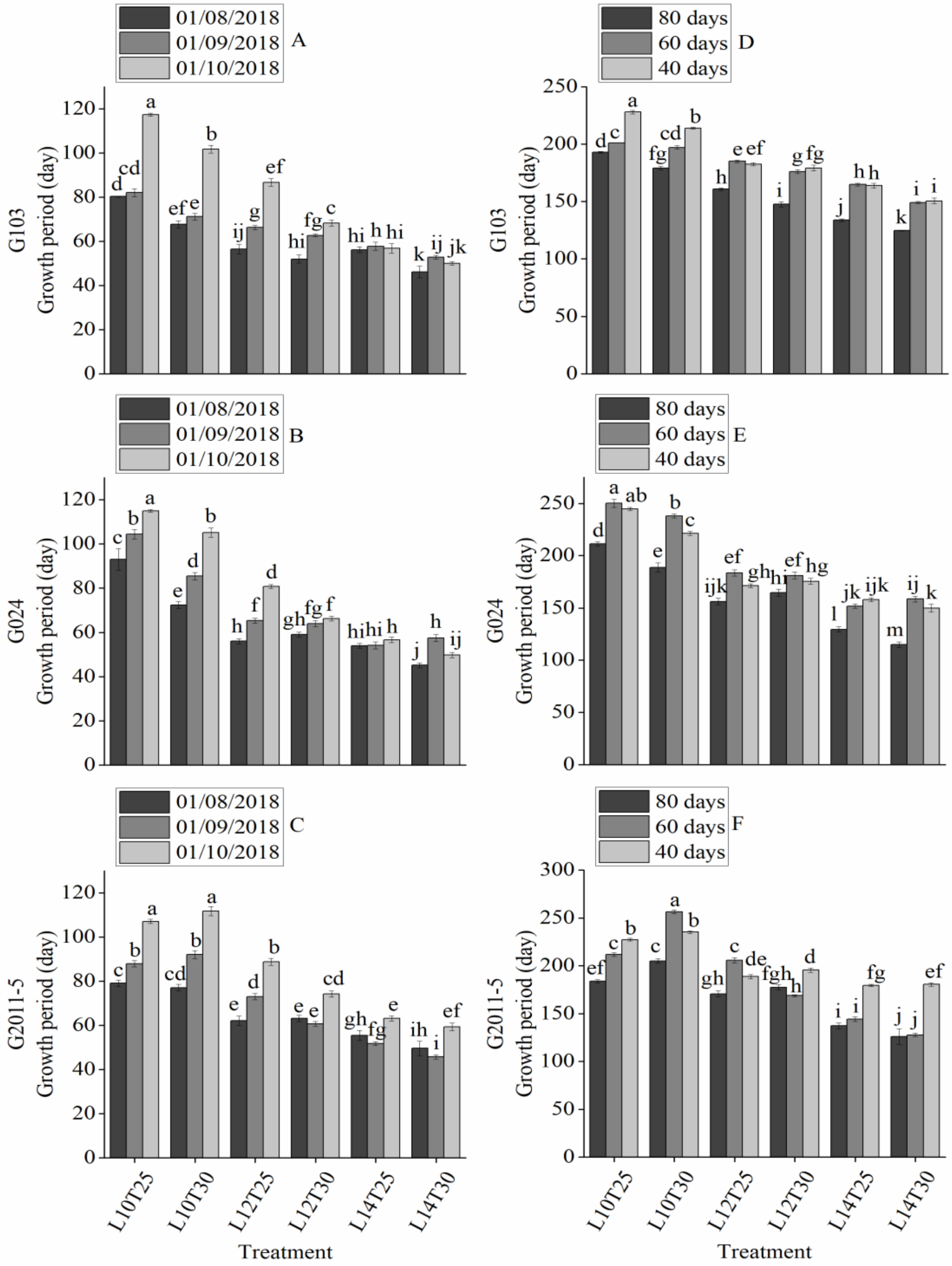

Figure S8. Growth period (day) of cvs. G103 (A), G024 (B) and G2011-5 (C) sown on 1st August, 1st September and 1st October; cvs. G103 (D), G024 (E) and G2011-5 (F)- 80 days after planting, 60 days after planting and 40 days after planting. Different letters indicate significant differences between sowing date, plant age, photoperiod and temperature at $P<0.05$ (ANOVA and Tukey HSD test); means±SD

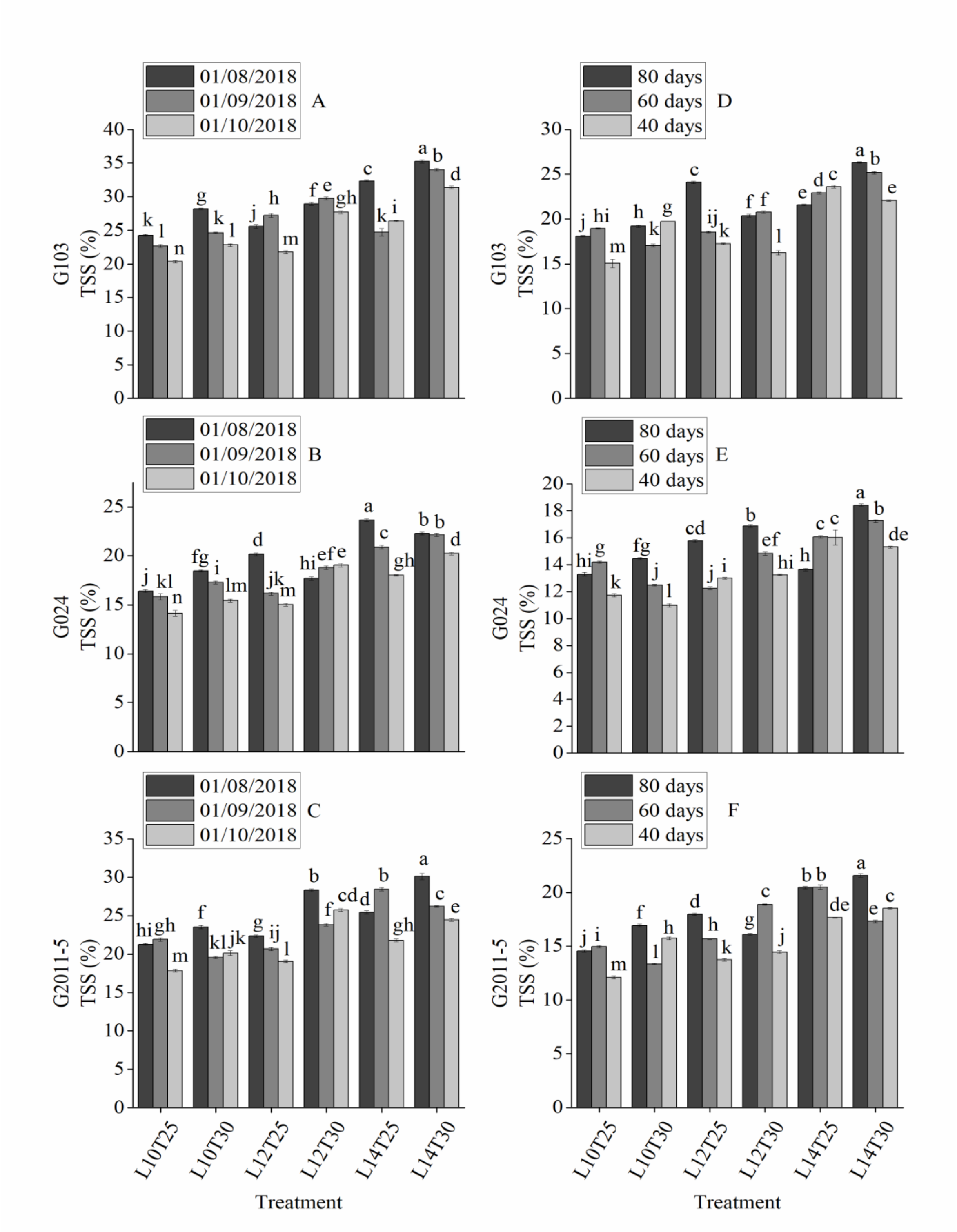

Figure S9. TSS (%) of cvs. G103 (A), G024 (B) and G2011-5 (C) sown on 1st August, 1st September and 1st October; cvs. G103 (D), G024 (E) and G2011-5 (F)- 80 days after planting, 60 days after planting and 40 days after planting. Different letters indicate significant differences between sowing date, plant age, photoperiod and temperature at $P<0.05$ (ANOVA and Tukey HSD test); means±SD

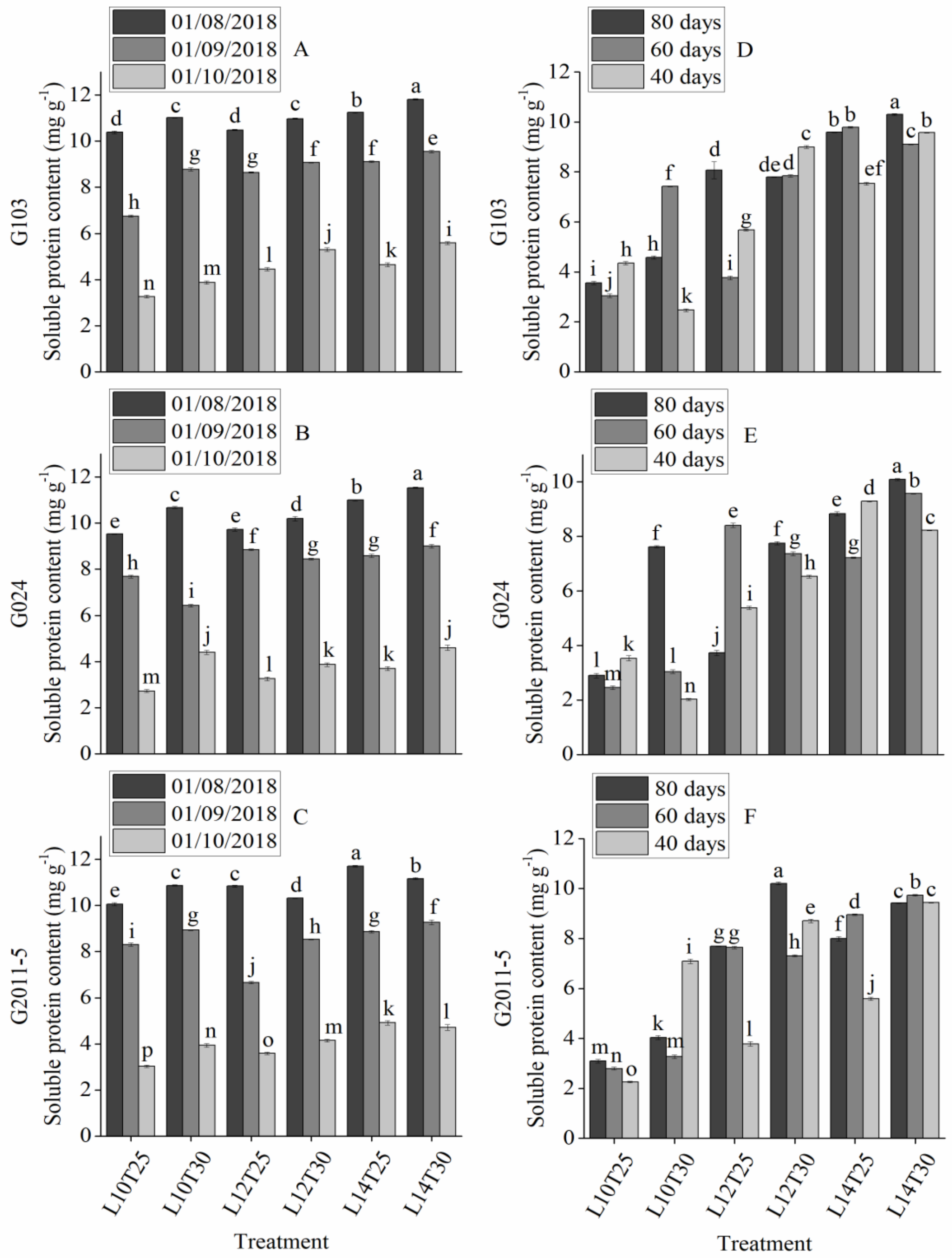

Figure S10. Soluble protein content (mg g^{-1}) of cvs. G103 (A), G024 (B) and G2011-5 (C) sown on 1st August, 1st September and 1st October; cvs. G103 (D), G024 (E) and G2011-5 (F)- 80 days after planting, 60 days after planting and 40 days after planting. Different letters indicate significant differences between sowing date, plant age, photoperiod and temperature at $P<0.05$ (ANOVA and Tukey HSD test); means±SD

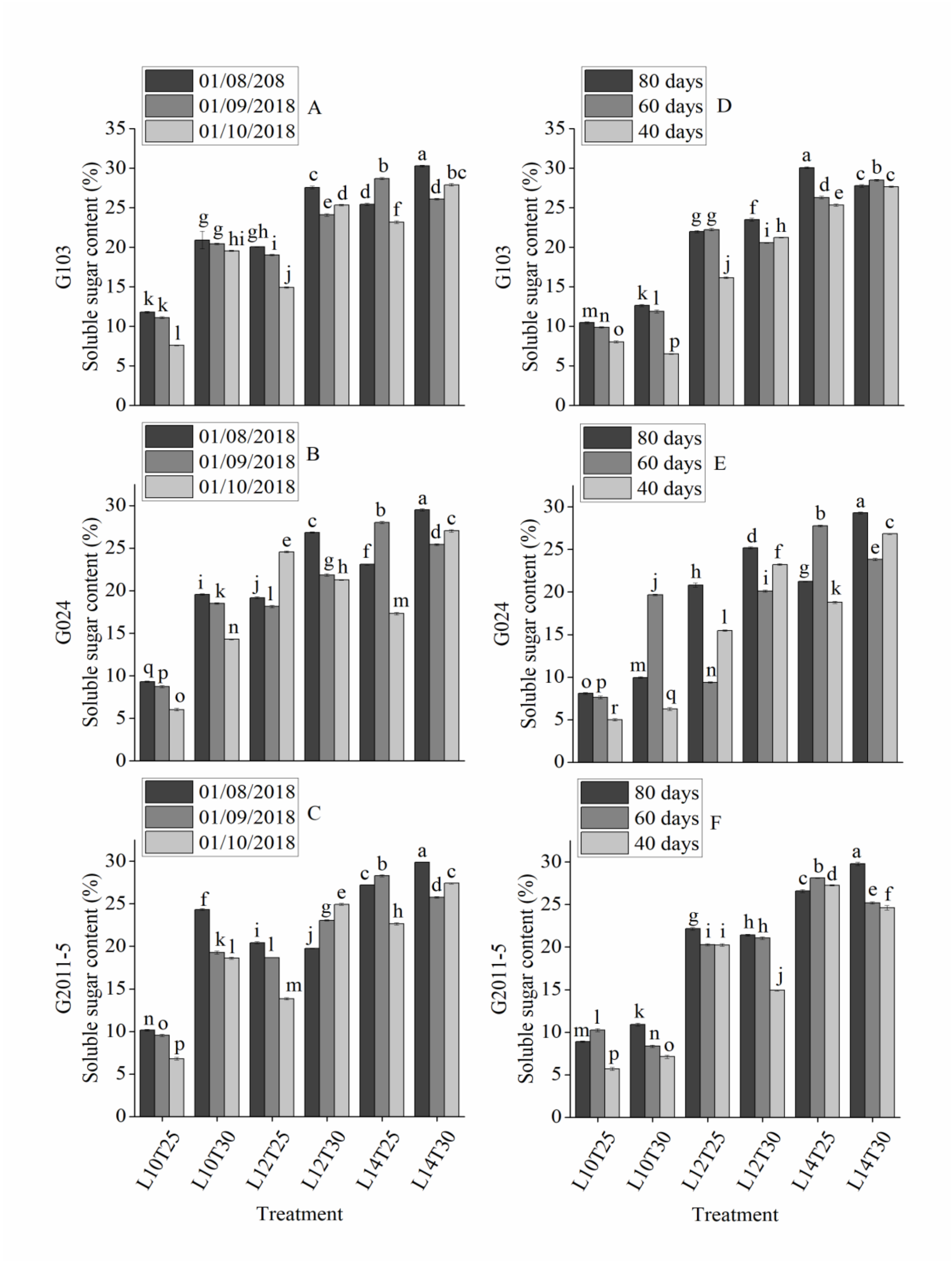

Figure S11. Soluble sugar content (%) of cvs. G103 (A), G024 (B) and G2011-5 (C) sown on 1st August, 1st September and 1st October; cvs. G103 (D), G024 (E) and G2011-5 (F)- 80 days after planting, 60 days after planting and 40 days after planting. Different letters indicate significant differences between sowing date, plant age, photoperiod and temperature at P<0.05 (ANOVA and Tukey HSD test); means±SD

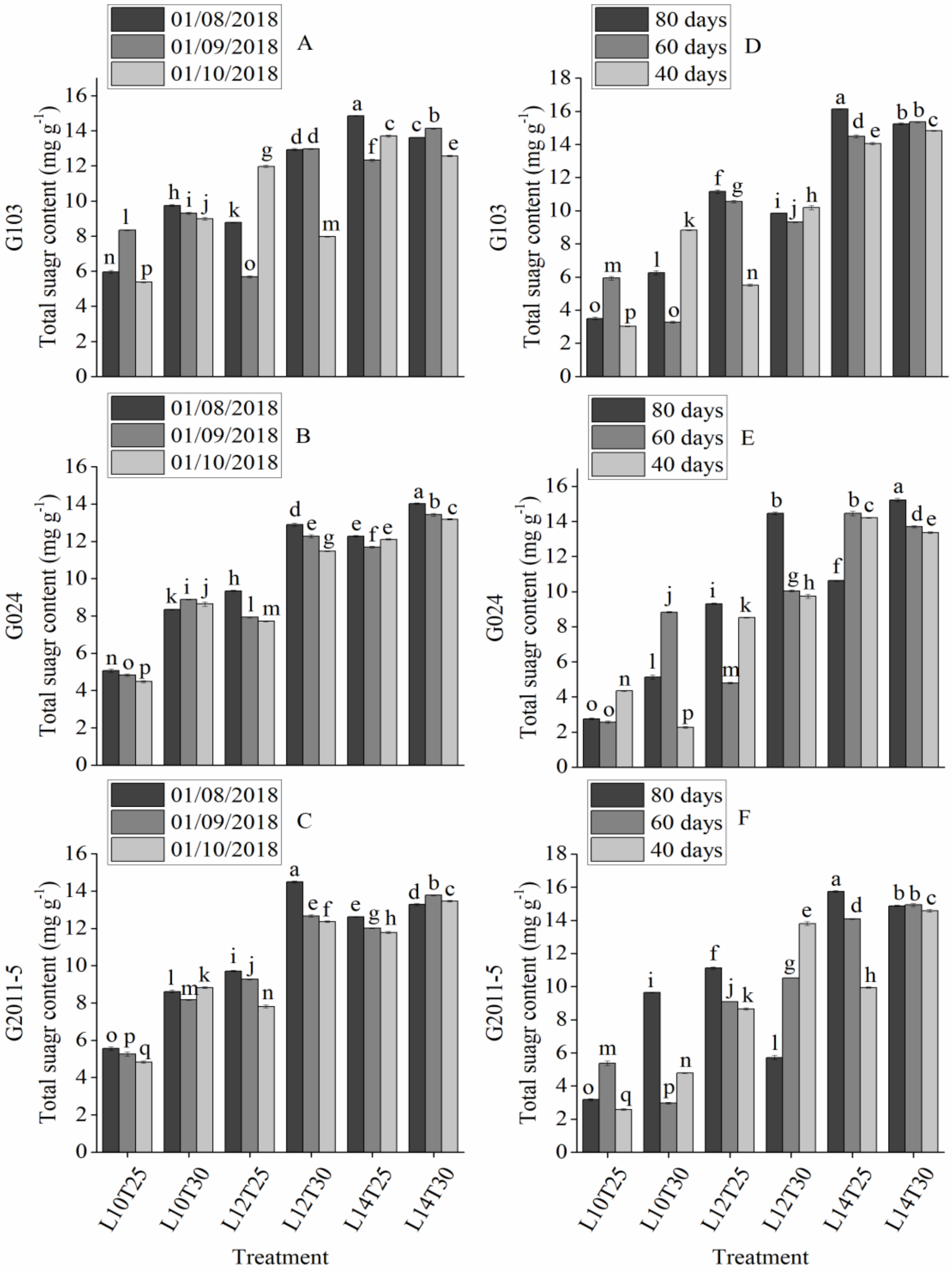

Figure S12. Total sugar content (mg g^{-1}) of cvs. G103 (A), G024 (B) and G2011-5 (C) sown on 1st August, 1st September and 1st October; cvs. G103 (D), G024 (E) and G2011-5 (F)- 80 days after planting, 60 days after planting and 40 days after planting. Different letters indicate significant differences between sowing date, plant age, photoperiod and temperature at $P<0.05$ (ANOVA and Tukey HSD test); means±SD

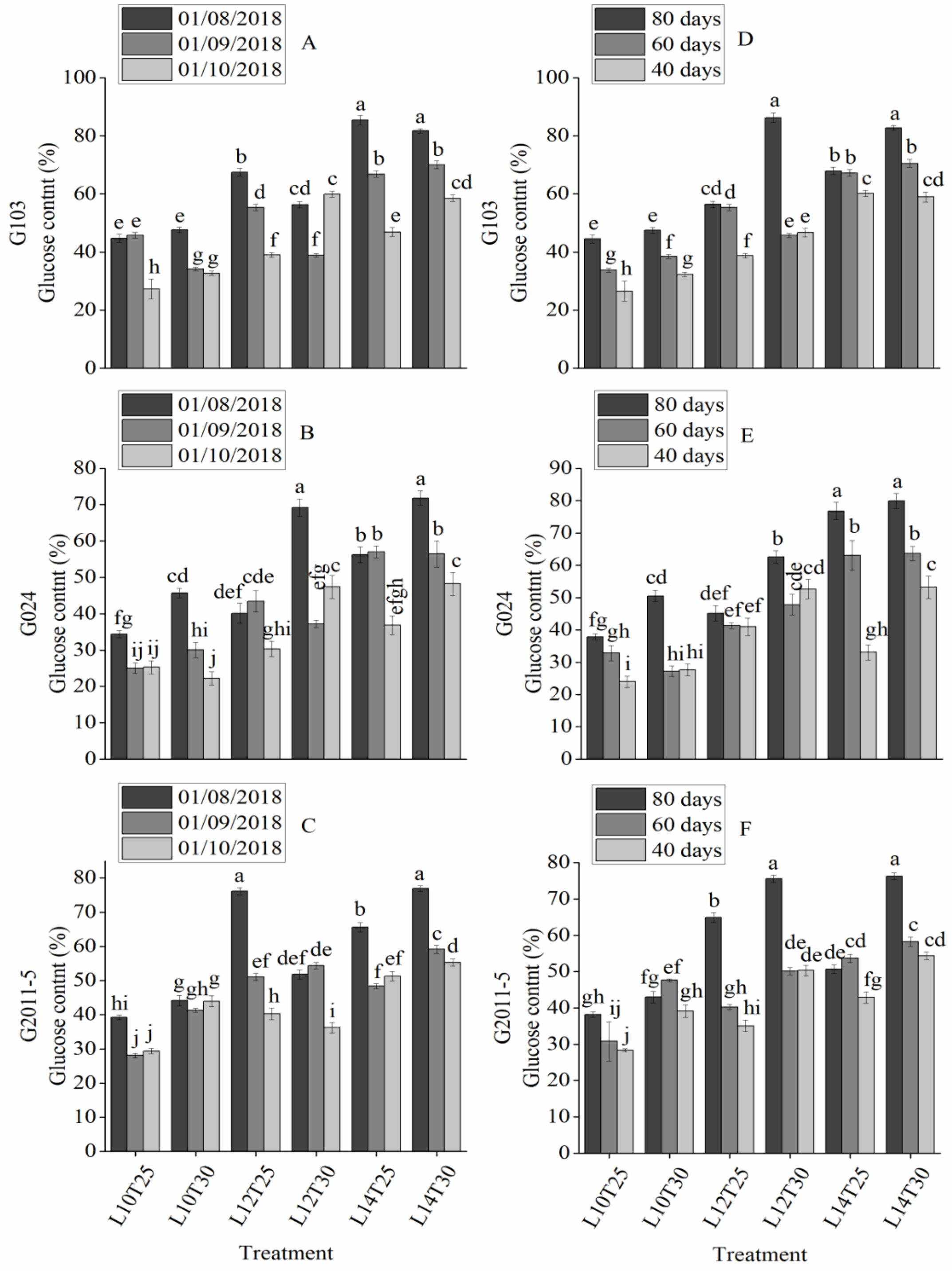

Figure S13. Glucose content (%) of cvs. G103 (A), G024 (B) and G2011-5 (C) sown on 1st August, 1st September and 1st October; cvs. G103 (D), G024 (E) and G2011-5 (F)- 80 days after planting, 60 days after planting and 40 days after planting. Different letters indicate significant differences between sowing date, plant age, photoperiod and temperature at P<0.05 (ANOVA and Tukey HSD test); means±SD

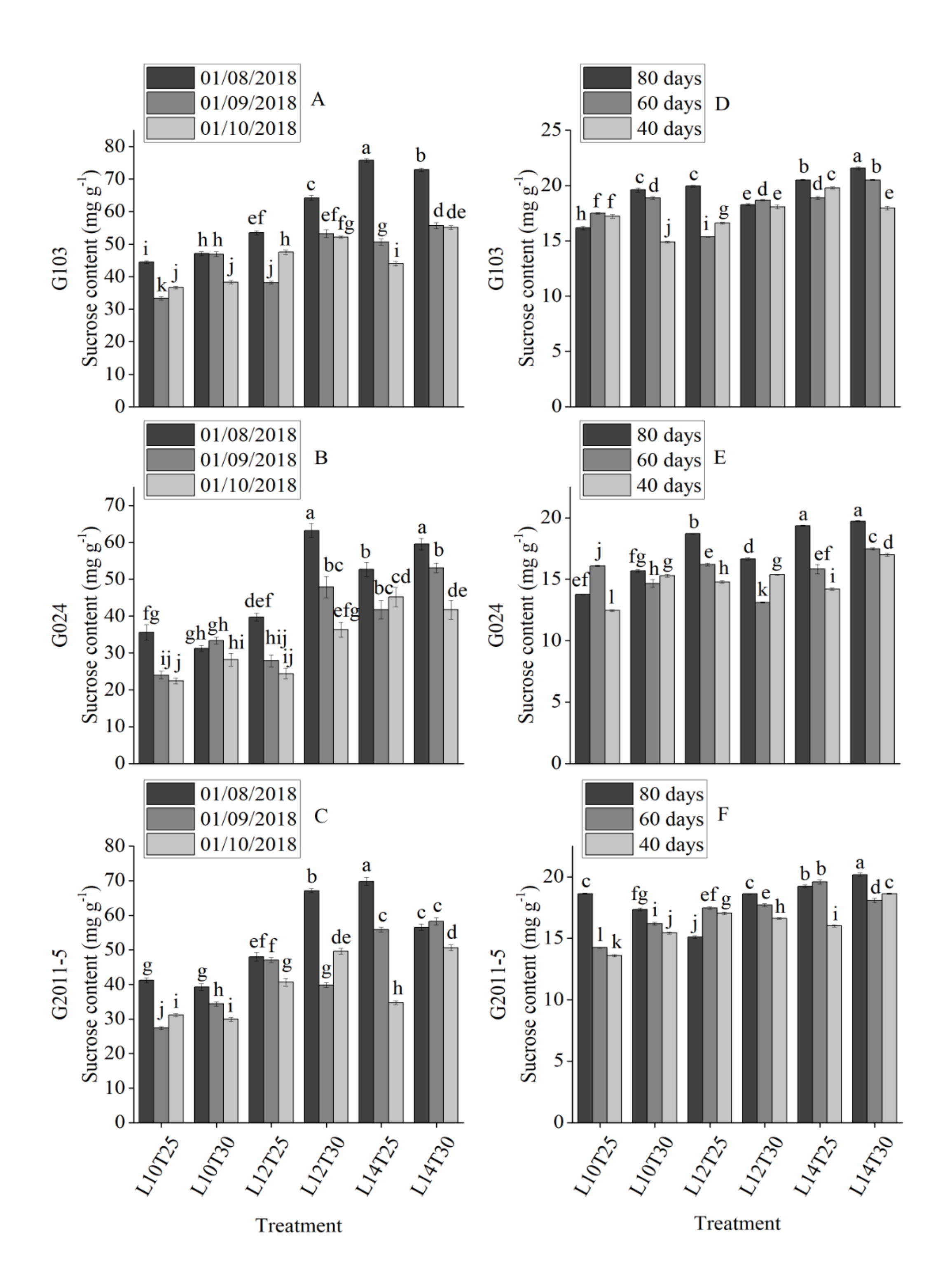

Figure S14. Sucrose content (mg g^{-1}) of cvs. G103 (A), G024 (B) and G2011-5 (C) sown on 1st August, 1st September and 1st October; cvs. G103 (D), G024 (E) and G2011-5 (F)- 80 days after planting, 60 days after planting and 40 days after planting. Different letters indicate significant differences between sowing date, plant age, photoperiod and temperature at P<0.05 (ANOVA and Tukey HSD test); means±SD

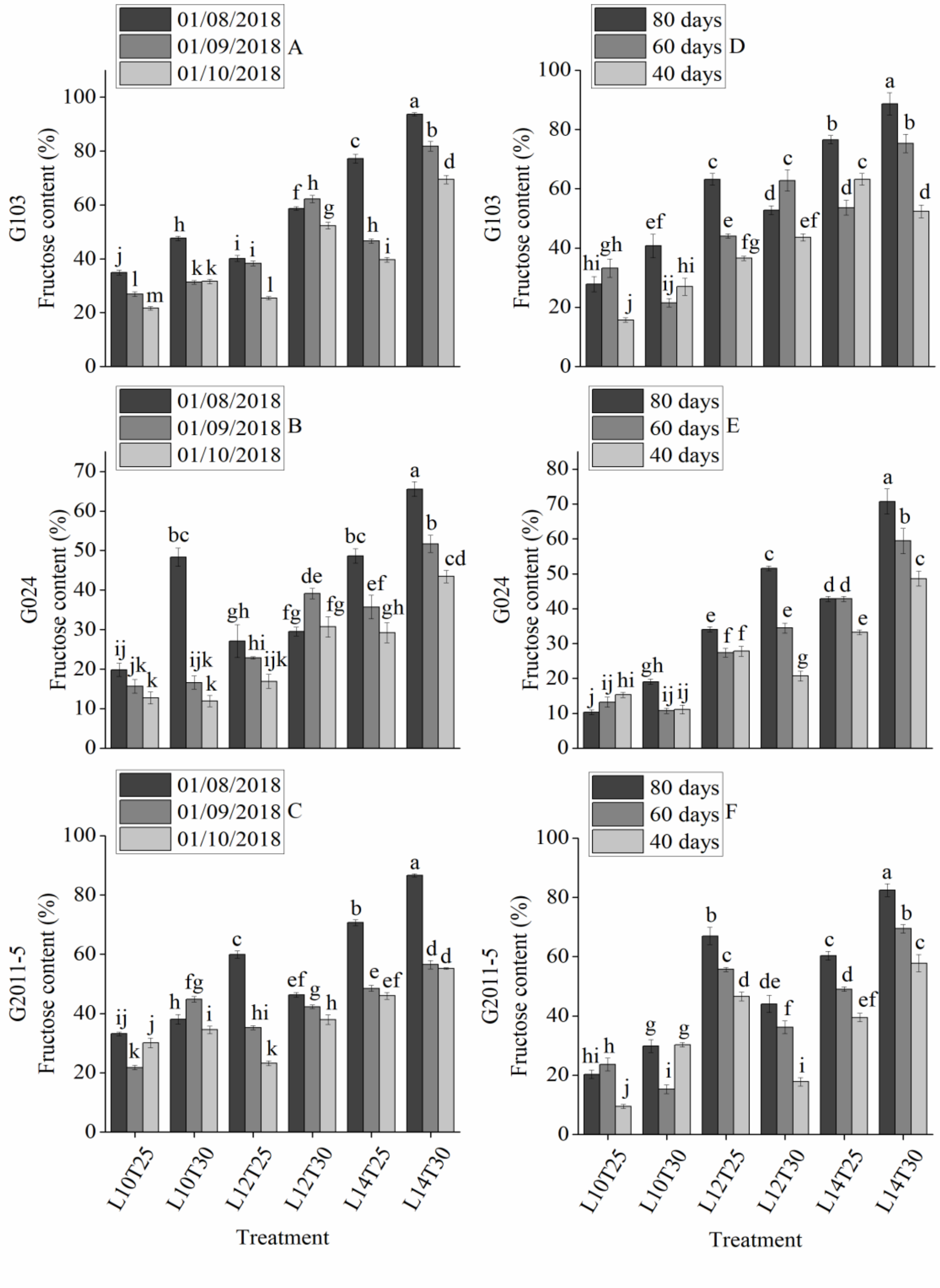

Figure S15. Fructose content (%) of cvs. G103 (A), G024 (B) and G2011-5 (C) sown on 1st August, 1st September and 1st October; cvs. G103 (D), G024 (E) and G2011-5 (F)- 80 days after planting, 60 days after planting and 40 days after planting. Different letters indicate significant differences between sowing date, plant age, photoperiod and temperature at $P<0.05$ (ANOVA and Tukey HSD test); means±SD

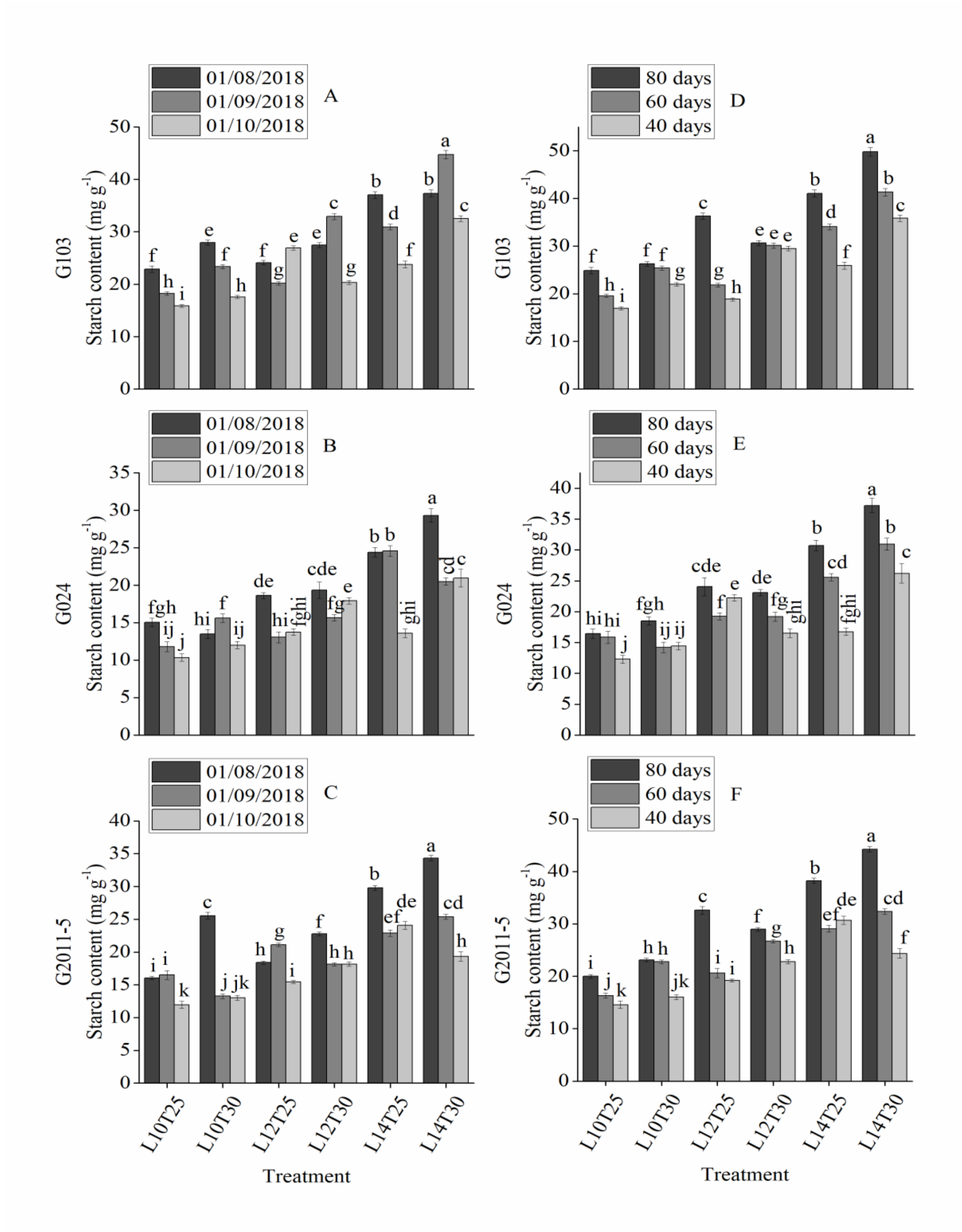

Figure S16. Starch content (mg g^{-1}) of cvs. G103 (A), G024 (B) and G2011-5 (C) sown on 1st August, 1st September and 1st October; cvs. G103 (D), G024 (E) and G2011-5 (F)- 80 days after planting, 60 days after planting and 40 days after planting. Different letters indicate significant differences between sowing date, plant age, photoperiod and temperature at P<0.05 (ANOVA and Tukey HSD test); means±SD

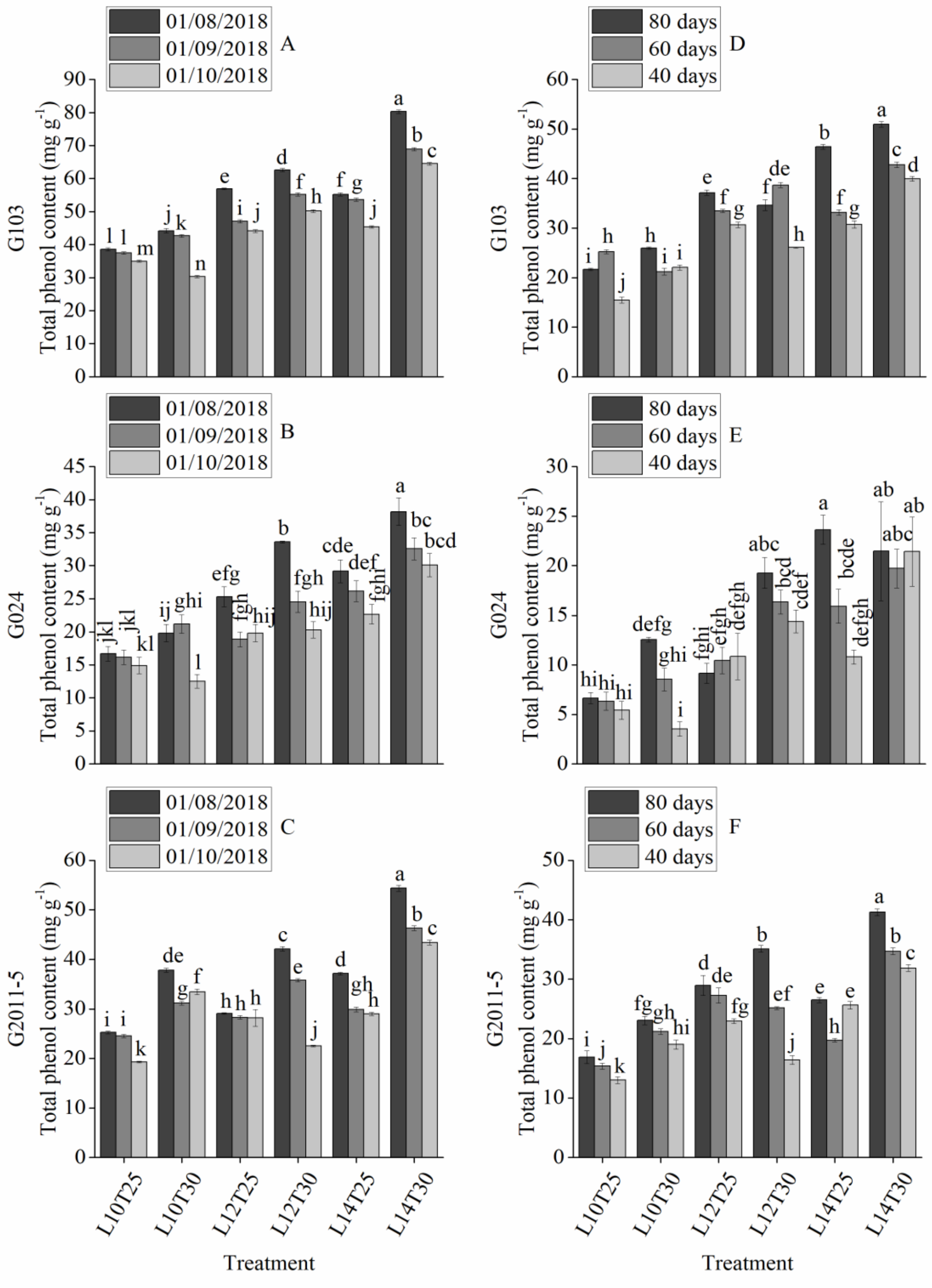

Figure S17. Total phenol content (mg g^{-1}) of cvs. G103 (A), G024 (B) and G2011-5 (C) sown on 1st August, 1st September and 1st October; cvs. G103 (D), G024 (E) and G2011-5 (F)- 80 days after planting, 60 days after planting and 40 days after planting. Different letters indicate significant differences between sowing date, plant age, photoperiod and temperature at P<0.05 (ANOVA and Tukey HSD test); means±SD

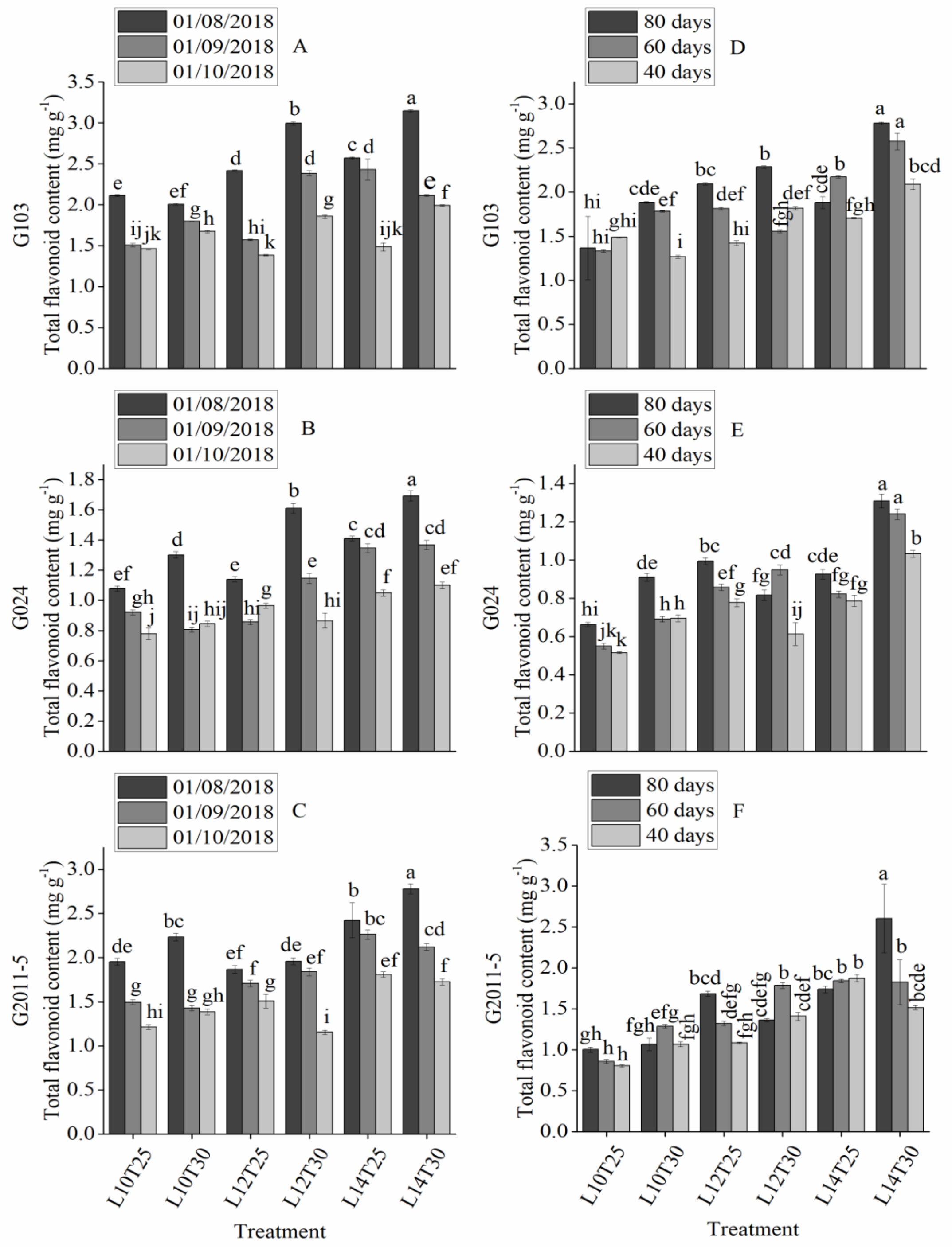

Figure S18. Total flavonoid content (mg g^{-1}) of cvs. G103 (A), G024 (B) and G2011-5 (C) sown on 1st August, 1st September and 1st October; cvs. G103 (D), G024 (E) and G2011-5 (F)- 80 days after planting, 60 days after planting and 40 days after planting. Different letters indicate significant differences between sowing date, plant age, photoperiod and temperature at $P<0.05$ (ANOVA and Tukey HSD test); means±SD
